# Identification of quantitative trait nucleotides and candidate genes for tuber yield and mosaic virus tolerance in an elite population of white guinea yam (*Dioscorea rotundata*) using genome-wide association scan

**DOI:** 10.1186/s12870-021-03314-w

**Published:** 2021-11-22

**Authors:** Paterne A. Agre, Prince E. Norman, Robert Asiedu, Asrat Asfaw

**Affiliations:** 1 International Institute of Tropical Agriculture, PMB 5320, Oyo Road, Ibadan, Oyo State 200001 Nigeria; 2 Sierra Leone Agricultural Research Institute, PMB 1313, Tower Hill, Freetown, Sierra Leone

**Keywords:** Genetic diversity, population structure, GWAS, SNP markers, white Guinea yam

## Abstract

**Background:**

Improvement of tuber yield and tolerance to viruses are priority objectives in white Guinea yam breeding programs. However, phenotypic selection for these traits is quite challenging due to phenotypic plasticity and cumbersome screening of phenotypic-induced variations. This study assessed quantitative trait nucleotides (QTNs) and the underlying candidate genes related to tuber yield per plant (TYP) and yam mosaic virus (YMV) tolerance in a panel of 406 white Guinea yam (*Dioscorea rotundata*) breeding lines using a genome-wide association study (GWAS).

**Results:**

Population structure analysis using 5,581 SNPs differentiated the 406 genotypes into seven distinct sub-groups based delta K. Marker-trait association (MTA) analysis using the multi-locus linear model (mrMLM) identified seventeen QTN regions significant for TYP and five for YMV with various effects. The seveteen QTNs were detected on nine chromosomes, while the five QTNs were identified on five chromosomes. We identified variants responsible for predicting higher yield and low virus severity scores in the breeding panel through the marker-effect prediction. Gene annotation for the significant SNP loci identified several essential putative genes associated with the growth and development of tuber yield and those that code for tolerance to mosaic virus.

**Conclusion:**

Application of different multi-locus models of GWAS identified 22 QTNs. Our results provide valuable insight for marker validation and deployment for tuber yield and mosaic virus tolerance in white yam breeding. The information on SNP variants and genes from the present study would fast-track the application of genomics-informed selection decisions in breeding white Guinea yam for rapid introgression of the targeted traits through markers validation.

**Supplementary Information:**

The online version contains supplementary material available at 10.1186/s12870-021-03314-w.

## Background

Root and tuber crops are significant contributors to global food supply next to cereal crops. Yam is among the principal root and tuber crops, after cassava and potato, that are widely grown and consumed as subsistence staples [1]. Yam is a collective name for the *Dioscorea* species extensively cultivated in the tropics and subtropics by smallholder farmers for its starchy underground tuber and aerial bulbils [2, 3]. The global estimated mean annual yam production and gross values are approximately 73 million tons and 14 billion US dollars, respectively, with West Africa accounting for 92% of the total yam production [4, 5]. There are over 600 *Dioscorea* species, of which 11 are economically significant [6]. White Guinea yam (*D. rotundata*), indigenous to Africa, is the most produced and consumed among cultivated species, supporting the livelihood of over 300 million people [2]. Yam is also important in many key life ceremonies in the major producing areas of West Africa [7].

Despite its socio-economic importance, a significant yield increase has not been achieved over the decades compared to cereal crops [1]. Improved varieties are vital for attaining increased productivity in farmers’ fields. The development of improved yam varieties requires a better understanding of the genetic control of traits contributing to the increased yield and acceptable quality by growers and consumers. However, the breeding efforts have not adequately explored the genetic basis of tuber yield and virus resistance traits to fast-track improved cultivar development. Genes controlling key traits such as resistance to pests and diseases, tuber yield, and tuber quality traits exhibit quantitative inheritance. They may not be linked in a preferred direction, making improving these traits challenging using conventional breeding techniques [8]. In QTL mapping studies, the variation in virus resistance is attributed to a single major locus with a modest contribution [9]. Two random amplification of polymorphic DNA (RAPD) markers tightly linked in the coupling phase with Ymv-1 locus on the same linkage group were reported in resistant genotypes of *D. rotundata*.

For tuber yield, limited knowledge exists regarding QTL mapping studies [8]. The QTLs detected for YMV in yam were mainly based on conventional family-based linkage mapping. In contrast, the GWAS strategy using naturally occurring variants is a more robust and efficient method for identifying significant loci and the genes involved in the genetic control of complex traits. The GWAS strategy has increasingly been utilized in many crops, including root and tuber crops, to dissect the underlying genetic control mechanism in complex traits. However, GWAS mapping for tuber yield and YMV tolerance in yam has not been reported to date.

Supporting yam breeding efforts based on quantitative genetics principles and genomics tools is indispensable to increase the program’s effectiveness for increasing productivity. Yam cultivar development using conventional strategies spans at least ten years from crossing to variety release recommendation [4, 6]. The complementation of the traditional breeding techniques with advanced molecular tools has reduced the breeding cycle in crops [10]. In theory, genotypic information from molecular markers, when associated with phenotypic traits of interest, may be extensively used to select individuals with higher genetic value through marker-assisted selection (MAS) [11].

This study's objective was to dissect the genetic control of tuber yield and YMV tolerance in white Guinea yam.

## Material and methods

### Plant materials

The study panel comprised 406 white Guinea yam clones, of which, 36 were trait progenitors, 49 elite clones, and 321 early generation breeding lines from the IITA's yam breeding program (Supplementary Table 1). All the genotypes are from the International Institute of Tropical Agriculture, IITA Ibadan Nigeria and are maintained by the Yam Breeding Improvement Unit.

### Phenotyping

Phenotypic data on tuber yield per plant (TYP) and yam mosaic virus (YMV) severity were recorded on the plant materials assessed at different breeding stages at IITA in Nigeria. The TYP and YMV severity were recorded on plants in the field using the procedure described in yam ontology (http://www.cropontology.org/ontology/CO_343/Yam) and yam standard operation protocol [12]. Tuber yield was recorded in kilogram on a plant basis at harvest (eight months after planting). The YMV severity score was assessed at 30-day intervals from 2 to 6 months after planting based on a visual assessment of the relative area of plant leaf surfaces affected by the mosaic virus disease using a five-ordinal scale of 1–5. A score of 1 represented no visible symptoms of virus infection, 2 for mild mosaic, vein-banding, green spotting or flecking, curling and mottling on few leaves but no leaf distortion, 3 for low incidence (25–50%) of the mosaic virus on the entire plant, 4 for the severe mosaic on most leaves and leaf distortion, and 5 for severe mosaic and bleaching with severe leaf distortion and stunting. The virus severity score values were converted to percentages and then used to estimate the area under disease progress curve (AUDPC) values as described by Forbes et al. [13]:

$$\mathbf{AUDPC}={\sum}_{\boldsymbol{i}=\mathbf{1}}^{\boldsymbol{n}-\mathbf{1}}\left(\frac{{\boldsymbol{y}}_{\boldsymbol{i}}+{\boldsymbol{y}}_{\mathbf{i}+\mathbf{1}}}{\mathbf{2}}\right)\ \left({\boldsymbol{t}}_{\boldsymbol{i}+\mathbf{1}}-{\boldsymbol{t}}_{\boldsymbol{i}}\right)$$

where *y*_*i*_ = disease severity at the i^th^ observation, *t*_*i*_ = time (days) at the i^th^ observation, and n = total number of observations.

### Phenotypic data analysis

We applied a one-step linear mixed model that used G-matrix to compute the best linear unbiased predictor (BLUP) values of an individual clone for a trait from the best fit model using the average information criterion (AIC) in restricted maximum likelihood (REML) algorithm [14] in the ASReml-R version 4 package [15]. The model used was:

$${\boldsymbol y}_{\boldsymbol i\boldsymbol j}=\mathbf\pi+{\mathbf\beta}_{\mathbf i}+{\mathbf\tau}_{\boldsymbol j}+{\boldsymbol\gamma}_{\boldsymbol k}+{\mathrm\varepsilon}_{\boldsymbol i\boldsymbol j}+{\mathbf Z}_{\boldsymbol u}\mathbf u$$

where ***y***_***ij***_ is the phenotypic value, μ is the overall average (shared by all observations), β_***i***_ is the effect of block i, τ_***j***_ is the specific effect to genotype j, γk is the specific effect to trials k and ℇ_***ij***_ is an effect specific to each experimental unit (combination block and genotype ) and **Z**_***u***_**u** is the the vectors of random additive and non-additive genetic within location effects, respectively, with corresponding design matrix Zu. Accordingly, the genetic variance was partitioned into the additive effects, which were associated with a covariance structure proportional to genetic relationships derived from the molecular markers and the non-additive genetic effect. The non-additive genetic variance is explained by individual identity rather than the genomic relationship matrix following the approach described by Borgognone et al. [16] and Ovenden et al. [17].

Broad sense heritability (H^2^) estimates for the traits were calculated from phenotypic variance (σ^2^p) and the genotypic variance (σ^2^g). The BLUP values of the genotypes for the traits extracted from the best fit model were used as input for the GWAS model.

### Genotyping and SNP data analysis

For each genotype, total genomic DNA was isolated from lyophilized young and fully expanded healthy leaves. Deoxyribonucleic acid (DNA) was extracted from the leaf samples using the CTAB procedure with slight modification [18]. DNA quality and concentration were assessed using agarose gel and nanodrop, respectively, following the methods described in Aljanabi and Martinez [19]. High-throughput genotyping was conducted in 96 plex DArTseq protocol, and SNPs were called using the DArT's proprietary software, DArTSoft, as described by Killian et al. [20]. Reads and tags found in each sequencing result were aligned to the *Dioscorea rotundata* reference genome version 2 (https://drive.google.com/drive/folders/1H5T4xjKAEl9LliR-4qK_IR6TypCDe8nj) with Hisat2 [21]. The raw HapMap file generated was first converted to a Variant Call Format (VCF) and filtered for missing value and polymorphic SNPs using quality control criteria of low sequence depth <5; SNP markers with missing values >20%; minor allele frequency (MAF) <0.05 and heterozygosity >50. Of the 16,242 SNP markers subjected to the filtering quality criteria, 5,581 good-quality SNPs were retained for various analyses.

### Population genetic analysis

Various population genetic analysis methods were conducted to explore the structure and level of genetic diversity in the study material. The SNP distribution and the density were estimated using the ‘*Cmplot’* function implemented in the CMplot R package [22]. For the SNP mutation from the reference to the alternative, SNPlay open website was used to estimate the rate of the transition and transversion across the retained SNP. Statistics such as the minor allele frequency (MAF), the observed and the expected heterozygosity, and the polymorphism information content were estimated using the function "--freq" and "--hardy" using PLINK V1.90 [23].

The genetic relationship among the plant materials was explored using the principal component analysis (PCA) in FactorMiner R package [24]. For the PCA, the origin of the plant (early generation and parental profile) was used as factor.

Structure software version 2.3.3 [25, 26] was used to cluster samples into populations. Structure simulations were carried out using an admixture model with a burn-in period of 20000 iterations and a Markov chain Monte Carlo (MCMC) set at 20000. The simulations were repeated 3 times for K-values of 1 to 10. The optimal subpopulation model was investigated in several ways: (1) by applying the informal pointers (i.e. geographical origin) proposed by Pritchard et al. [25] and Falush et al. [27]; (2) by considering ΔK, a second order rate change with respect to K, as defined in Evanno et al. [28], as implemented in STRUCTURE HARVESTER [29] and thus the most likely value of K determined. Structure population was then plotted using barplot function implemeneted in R. The phylogeny tree was done using ape version 5.0 implemented in R [30].

### Genome Wide-Association Analysis (GWAS)

The GWAS were performed using the R package mrMLM v4.0.2 [31] with six multi-locus models. These models included: 1) multi-locus random-SNP-effect Mixed Linear Model [32], 2) Fast multi-locus random-SNP-effect EMMA (FASTmrEMMA) [33], 3) Iterative Sure Independence Screening EM-Bayesian LASSO (ISIS EM-BLASSO) [34], 4) polygenic-background-control- based least angle regression plus empirical Bayes (pLARmEB) [35], 5) polygenic- background-control-based Kruskal-Wallis test plus empirical Bayes (pKWmEB) [36]; and 6) fast mrMLM (FASTmrMLM) [37].

In the mrMLM analysis, we accounted for population structure (Q) generated from Structure analysis. For each trait, the optimal number Q value included in the GWAS models was determined based on the highest ΔK value. The percentage of variation explained by the associated marker (R^2^) and the markers effect were estimated in the mrMLM (v 4.0.2) R package (https://cran.r-project.org/web/packages/mrMLM/index.html).

### Identification of existing putative genes

The possible candidate genes within the significant QTL region were searched in the defined range window of 1 MB at 500 Kb (downstream and upstream) from the yam Generic File Format (GFF3) file. Linkage disequilibrium (LD) was assessed between the significant SNPs using the LDheatmap library [38]. The yam generic feature format (GFF3) of the reference genome was used to identify the main gene in the inter-genic region using the SNPReff. Functions of the genes associated with the identified SNPs were determined using the public database Interpro, European Molecular Biology Laboratory-European Bioinformatics Institute (EMBL-EBI) [39].

### Haplotype estimation and SNP markers effect prediction

Haplotype associated with significant QTL was developed using “*rstatix”* package implemented in R, and the sequence of each haplotype was defined based on the 406 genetic material considered as testing and or identification population. The variant effect prediction was evaluated through the adjusted posterior probability, and the markers with high segregation were identified. Marker effects were then plotted for vizualization.

## Results

### Phenotypic data of the white yam

Table 1 presents summary statistics for the phenotypic traits assessed. Broad-sense heritability estimates were high, 0.708 for tuber yield per plant and 0.903 for yam mosaic virus. The phenotypic value for the tuber yield ranged from 0.93 to 1.47 kg plant^-1^ with an average of 1.19 kg. The area under the disease progress curve for YMV ranged from 100.56 to 2900.45 with an average of 936.16. (Supplementary Table 2).

**Table 1:** Descriptive statistics of tuber yield per plant (TYP) and yam mosaic virus (YMV)

Traits	Minimum	Maximum	Mean	Standard Deviation	Broad sense heritability (H^**2**^)
TYP	0.93	1.47	1.19	0.11	0.708
YMV	100.56	2900.45	936.16	481.19	0.903

*TYP* Tuber yield per plant, *YMV* yam mosaic virus (AUDPC value)

### Genetic diversity, population structure and linkage disequilibrium

The DArT genotyping of 406 white Guinea yam clones detected the highest number of SNPs (637) mapped on chromosome 5 and the lowest of 123 on chromosome 11 (Supplementary Fig. 1A). Transition SNPs (60.13%, 3,356 SNPs) were more frequent than transversions (39.87%, 2225 SNPs) (Supplementary Fig. 1B). The observed heterozygosity value ranged from 0.029 to 0.622, with an average of 0.336 (Supplementary Fig. 1C). The expected heterozygosity value ranged from 0.09 to 0.5, with an average of 0.331 (Supplementary Fig. 1D). The minor allele frequency ranged from 0.05 to 0.5, with a mean of 0.24 (Supplementary Fig. 1E). The polymorphic information content (PIC) ranged from 0.087 to 0.335, with an average value of 0.267 (Supplementary Fig. 1F).

**Fig. 1:**
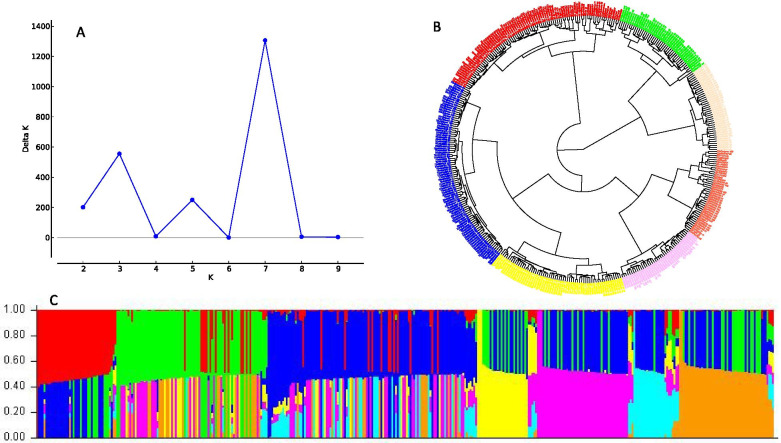
Graphical representation of the population structure of the 406 yam diversity panel. **A** Plot of mean likelihood of delta K against the number of K groups. The highest peak observed at K=7 signifies the grouping of accessions into seven groups. **B** Phylogeny tree showing the 7 Sub-populations. The colors represent each sub-population. **C** Population structure originated from the STRUCTURE based K=7. Each vertical barplot represents a single yam clone

The population structure analysis of the yam diversity panel shows that the delta K values from the mean log-likelihood probabilities plateaued at K=7 (1306.47) (Fig. 1A). At K=7, the 406 yam diversity panel was divided into 7 sub-populations (Fig. 1C). Using the 50% cutoff criterion of membership probability threshold, 305 accessions were successfully assigned to the 7 different sub-populations. The remaining 101 accessions with a probability of associations less than 50% were designated as an admixed population. The phylogenetic tree also showed seven sub-populations with higher degrees of admixture similar to the delta K plot from the STRUCTURE (Fig. 1B).

**Fig. 2:**
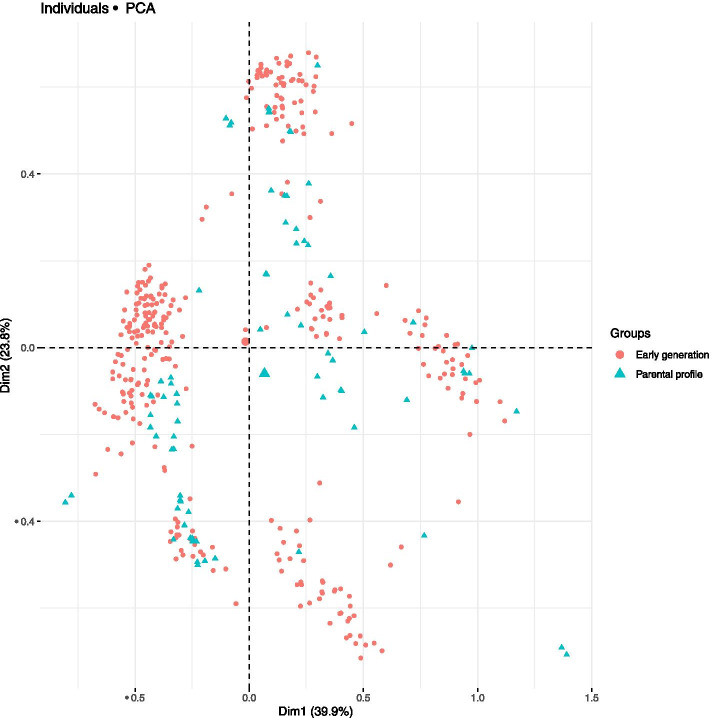
Principal component displaying the relationship between and among the early generation and parental profile clones used in this study

Exploring the the genetic relashionship through principal component analysis showed that the first two PCs account for 63.7% of the total variation (Fig. 2). The PCA clearly showed a higher degree of admixture between the early generation and parental profile clones. Both the early generation and the parental profile clones were distributed along PC1 and the PC2 (Fig. 2).

### Genome-wide scan for traits

#### Tuber yield

**Fig. 3:**
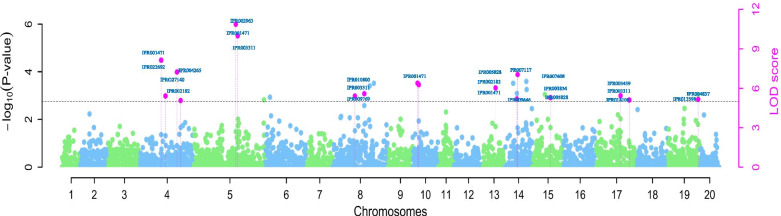
Genome-wide association analysis of tuber yield per plant. Manhattan plot indicating three SNP markers located on chromosomes 4, 5, 8, 10, 13, 14, 15, 17 and 19 associated with the tuber yield per plant. The blue letters are the Interpro ID for the different putative genes near the SNP markers associated with the tuber yield per plant

We found seventeen SNPs markers distributed on 9 chromosomes, significantly associated with tuber yield (kg plant^-1^) (Table 2; Fig. 3). The LOD values for these SNPs ranged from 5.07 to 10.88 with minor allele frequency (MAF) ranging from 0.09 to 0.50. Of the 17 SNP markers associated with tuber yield, four were mapped on chromosome 4, two on chromosome 5, two each on chromosomes 8, 10, 14, and 17 and a single SNP each on chromosomes 13, 15, and 19 (Table 2). The SNP marker chr05_24682916 explained the highest total phenotypic variance 8.47%.

**Table 2:** SNP markers associated with the tuber yield per plant (TYP) and yam mosaic virus severity score.

Trait	Method	SNP marker	Chr	pos (bp)	QTN effect	LOD score	‘-log10(P)’	r^2^ (%)	MAF	Genotype for code 1
YMV	pLARmEB	chr03_6338751	3	6338751	-143.86	6.10	6.93	4.68	0.46	T
	pKWmEB	chr05_30671001	5	30671001	-109.57	5.32	6.13	5.96	0.49	A
	pLARmEB	chr10_1116193	10	1116193	206.37	5.24	6.05	3.87	0.26	A
	pLARmEB	chr15_3906069	15	3906069	211.65	6.88	7.74	0.31	0.16	A
	pKWmEB	chr15_3906069	15	3906069	174.41	6.15	6.99	0.33	0.16	A
	pKWmEB	chr16_1482029	16	1482029	-100.04	5.00	5.80	3.29	0.49	T
TYP	mrMLM	chr04_23401186	4	23401186	-0.02	5.07	5.87	3.76	0.45	A
	pLARmEB	chr04_8196378	4	8196378	-0.03	5.42	6.23	0.43	0.17	T
	pLARmEB	chr04_18269860	4	18269860	-0.02	7.23	8.10	1.41	0.48	C
	pKWmEB	chr04_6236404	4	6236404	-0.03	8.14	9.03	5.25	0.25	T
	pLARmEB	chr05_24237388	5	24237388	-0.02	10.88	11.83	1.86	0.45	T
	pKWmEB	chr05_24682916	5	24682916	0.03	10.00	10.94	8.47	0.39	A
	pKWmEB	chr08_7046574	8	7046574	-0.01	5.41	6.23	7.38	0.21	A
	pKWmEB	chr08_10135940	8	10135940	-0.02	5.59	6.41	1.64	0.26	C
	pKWmEB	chr10_1571815	10	1571815	-0.03	6.28	7.12	0.86	0.15	C
	pKWmEB	chr10_1317508	10	1317508	-0.01	6.39	7.24	2.41	0.41	T
	FASTmrMLM	chr13_13467988	13	13467988	-0.02	6.04	6.87	2.93	0.41	T
	FASTmrMLM	chr14_11301309	14	11301309	-0.08	7.04	7.91	1.08	0.11	A
	pLARmEB	chr14_11128124	14	11128124	-0.04	5.19	5.99	1.78	0.15	G
	pLARmEB	chr15_5858214	15	5858214	0.02	5.30	6.10	0.39	0.32	T
	mrMLM	chr17_15363223	17	15363223	-0.06	5.44	6.25	0.01	0.10	T
	pLARmEB	chr17_19041958	17	19041958	-0.02	5.11	5.91	0.16	0.14	C
	pLARmEB	chr19_9446619	19	9446619	-0.03	5.16	5.96	0.70	0.09	G

#### Yam mosaic virus resistance

**Fig. 4:**
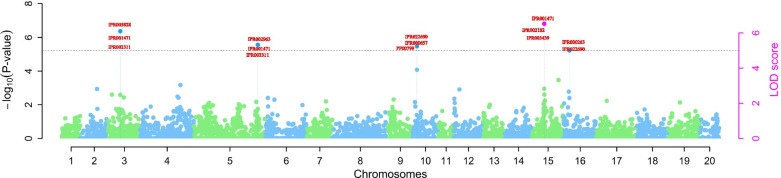
Genome-wide association analysis of yam mosaic virus. Manhattan plot indicating SNPs associated with the YMV. The *y-axis* represents the *p-value* of the marker-trait association on a –log_10_ scale. The red letters are the Interpro ID for the different putative genes near the SNP markers associated with the yam mosaic virus

We found five SNP loci that showed a significant association with the reaction to mosaic virus infection (Table 2, Fig. 4). Of the significant SNPs associated with YMV, three markers named chr03_6338751, chr05_30671001 and chr16_1482029 displayed negative quantitative trait nucleotide effects (Table 2). Using different genetic model for the SNP association SNP marker chr15_3906069 located on chromosome 15 was identified by two methods pLARmEB and pKWmEB. The total phenotypic variance explained by the markers associated with the yam mosaic virus varied from 0.33% to 5.96%. The minor allele frequency (MAF) of the associated SNP marker ranged from 0.16 to 0.49.

### SNP-trait association mapping

Four multi-locus models (MLMs) including FASTmrMLM, mrMLM, pKWmEB and pLARmEB detected a total of 22 QTNs across the 20 chromosomes of white yam for TYP and YMV traits (Table 2). Of the 22 QTNs, a total of 17 SNPs significantly associated with TYP. Among the 17 loci, two SNPs each were detected by FASTmrMLM and mrMLM; and seven SNPs each by pKWmEB and pLARmEB. These QTNs were distributed unevenly on 9 chromosomes (Table 2). Models pKWmEB and pLARmEB detected the highest number of 7 QTNs each. The 7 QTNs of model pKWmEB were detected on chromosomes 4, 5, 8 and 10, while those of model pLARmEB were detected on chromosomes 4, 5, 14, 15, 17 and 19.

For YMV, a total of five QTNs were detected by pLARmEB and pKWmEB and unevenly distributed on five chromosomes.

*TYP* tuber yield (kg plant^-1^), *YMV* Yam mosaic virus severity score (AUDPC value), *LOD* Logarithm of odds, *Chr* chromosomes, *Pos* position, *bp* base-pair, *MAF* Minor allele frequency, *r*^*2*^ r-square, *QTN* quantitative trait nucleotide

### Identification of existing putative genes

#### Tuber yield

**Fig. 5:**
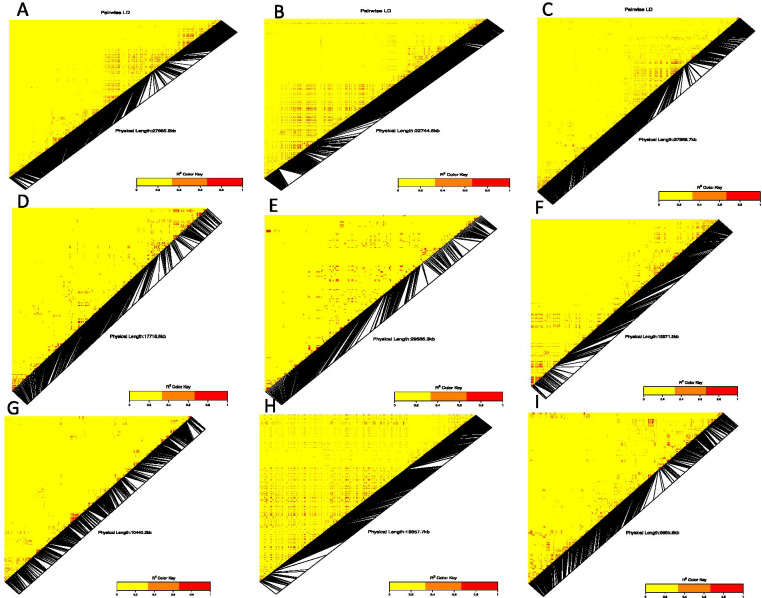
Heatmap LD haplotype blocks for different SNP markers located on different chromosomes **A** chromosome 4; **B** chromosome 5; **C** chromosome 8; **D** chromosome 10; **E** chromosome 13; **F** chromosome 14; **G** chromosome 15; **H** chromosome 17 and **I** chromosome 19. The R2 color key indicates the degree of significant association with the putative genes

We explored the association of the identified QTN regions on the physical map with the potential candidate genes and their functions using the white Guinea yam genome sequence. The LD heatmap of the significant SNPs on chromosomes 4, 5, 8, 13, 14, 15, 17 and 19 displayed a high genetic correlation (0.3 to 0.85) between the specific SNPs in the vicinity of the peak adjacent to the putative gene (Fig. 5). On chromosome 4, the significant SNP for tuber yield is located on the genomic regions harboring six putative genes (Gibberellin regulated protein, AP2/ERF domain, NB-ARC, Dirigent protein, Membrane transport protein, and Importin subunit beta-1, plants) with known functions. On chromosome 5, we detected three putative genes (Expansin, AUX/IAA protein and AP2/ERF domain). On chromosome 8, we identified two putative genes (AUX/IAA protein; Glycine-rich protein) (Supplementary Table 4). Several putative genes were identified on chromosome 14 (Supplementary Table S4). On chromosome 15, which displayed average correlation through the Ldheatmap, five genes were identified in the vicinity of the targeted SNP marker. The LD heatmap for the SNP found in association with tuber yield on chromosome 19 revealed the presence of 9 putative genes (ABC transporter-like, Exportin-1/Importin-beta-like, Sodium/calcium exchanger membrane region, AUX/IAA protein, Geminivirus AL3 coat protein, AP2/ERF domain, Major facilitator, sugar transporter-like, and Expansin).

#### Yam mosaic virus resistance

**Fig. 6:**
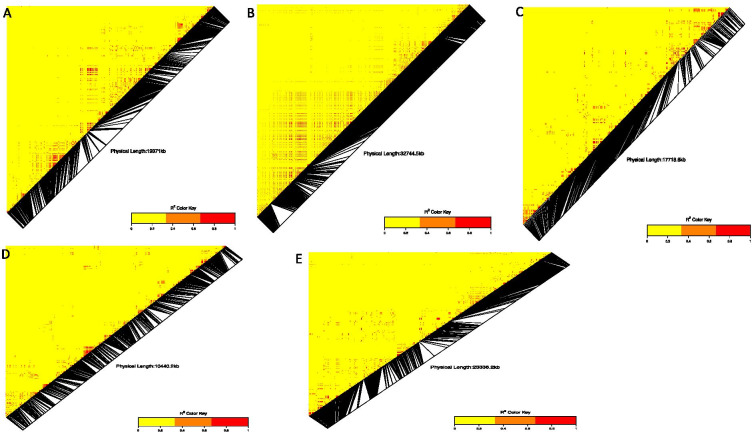
Summary of the local LD and haplotype blocks for different SNP marker located on different chromosome **A** chromosome 3, **B** chromosome 10, **C** Chromosome (5), **D** chromosome 15 and **E** chromosome 16 The R^2^ color key indicates the degree of significant association

We identified four candidate genes, namely AP2/ERF domain, Major facilitator, sugar transporter-like, and AUX/IAA protein on chromosome 3 near the SNP found in association with the YMV. The four identified candidate genes, AP2/ERF domain and AUX/IAA protein, were reported to confer essential gene functions related to plant defense and growth. The pairwise LD between the SNP of chromosome 3, 5, 10, 15 and 16 situated in genomic regions associated with YMV displayed a higher correlation with the three main haplotypes block (Fig. 6). On chromosome 10, fifteen different putative genes were identified near the significant SNPs as being associated with the YMV resistance, namely SNF2-related domain, Geminivirus AL3 coat protein, SANT/Myb domain, Geminivirus AL1 replication-associated protein, CLV type, Chlorophyll A-B binding protein, AP2/ERF domain, Gdt1 family, NB-ARC, Probable transposase, Ptta/En/Spm plant, Geminivirus AL1 replication-associated protein, catalytic domain, Kinesin-like protein and Geminivirus Rep catalytic domain.

### Haplotype SNP distribution and SNP markers effect prediction

**Fig. 7:**
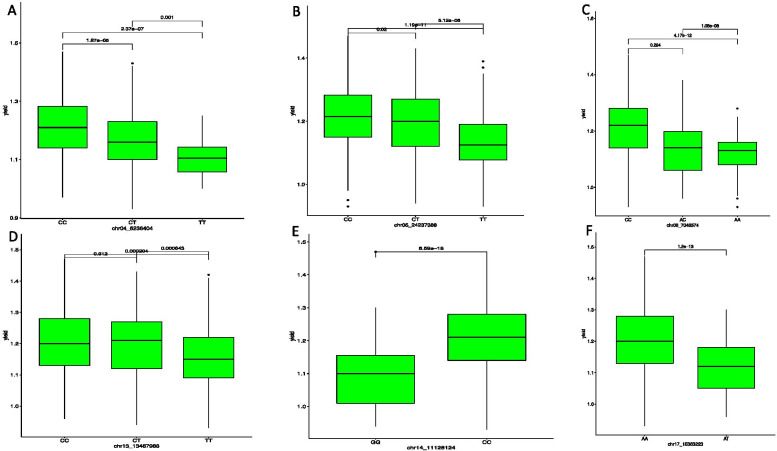
Boxplots showing the effect of the significant markers associated with tuber yield per plant on: **A** chromosome 4, **B** chromosome 5 **C** chromosome 8, **D** chromosome 13, **E** chromosome 14 and **F** chromosome 17. The letters on the X-axis represent allele variants

**Fig. 8:**
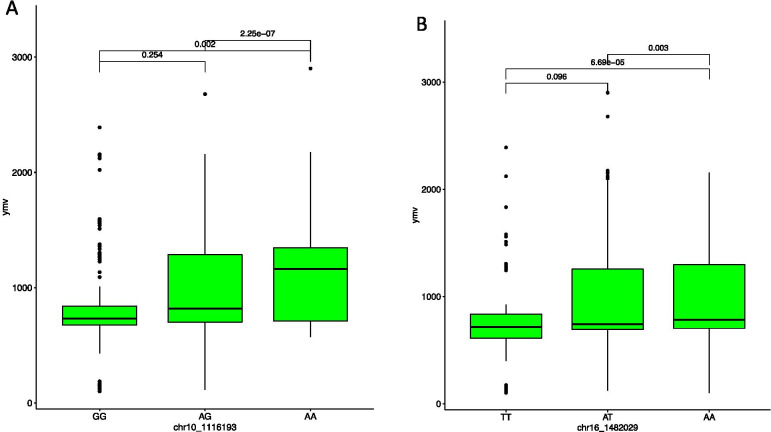
Boxplots showing the effect of the only significant markers associated with yam mosaic virus identified from the haplotype segragatio on: **A** chromosome 10 with chr10_1116193 (**B**) chromosome 16 with one SNP chr16_1482029. The letters on the X-axis represent allele variants for the different SNP markers

The frequencies and marker prediction effects of various haplotypes associated with tuber yield and resistance to yam mosaic virus in white Guinea yam are presented in Table 3. Of the seventeen SNP markers associated with the tuber yield, six SNP markers including chr04_6236404, chr05_24237388, chr08_7046574, chr13_13467988, chr14_11128124 and chr17_15363223 displayed high haplotype segregation among the different variants. Accordingly, the SNP markers on chromosomes 4, 5, 8, 13, 14 and 17 identified variants CC and CT to be associated with genotypes with higher tuber yield, whereas variants TT and AT were found to be associated with lower tuber yield (Fig. 7). Of the five SNP markers associated with the YMV, two (chr10_1116193 and chr16_1482029) were found to have high significant haplotype variations (Table 3). On chromosome 10, SNP markers associated with the YMV located at 1116193 bp showed that variants GG and AG were linked to lower predicted YMV value, while variant AA was identified to predict the higher YMV score (Fig. 8A). For the marker chr16_1482029 associated with YMV located at 1482029 bp variants TT and AT were linked to lower predicted YMV value (Fig. 8B).

**Table 3:** Frequencies and marker prediction effects of various haplotypes associated with tuber yield (kg plant^-1^) and reaction to yam mosaic virus infection (AUDPC value)

Traits	Markers	Hap	Seq	Freq	Adjusted probability	Prob. Adj. significance
Yield	chr04_6236404	Hap1	CCCT	0.475	3.74 e^-05^	****
		Hap2	CCTT	0.296	7.11 e^-07^	****
		Hap3	CTTT	0.228	0.001	***
	chr04_8196378	Hap1	CCCT	0.481	0.218	ns
		Hap2	CCTT	0.364	0.041	*
		Hap3	CTTT	0.154	0.218	ns
	chr04_18269860	Hap1	AAAC	0.328	0.399	ns
		Hap2	AACC	0.359	0.814	ns
		Hap3	ACCC	0.312	0.814	ns
	chr04_23401186	Hap1	AAAG	0.274	0.029	*
		Hap2	AAGG	0.396	0.001	***
		Hap3	AGGG	0.330	0.619	ns
	chr05_24237388	Hap1	CCCT	0.367	0.020	*
		Hap2	CCTT	0.316	3.57 e^-11^	****
		Hap3	CTTT	0.317	1.02 e^-05^	****
	chr05_24682916	Hap1	AAAC	0.295	0.921	ns
		Hap2	AACC	0.305	0.044	*
		Hap3	ACCC	0.400	0.043	*
	chr08_7046574	Hap1	AAAC	0.142	0.294	ns
		Hap2	AACC	0.423	1.25 e^-11^	****
		Hap3	ACCC	0.435	2.16 e^-08^	****
	chr08_10135940	Hap1	CCCG	0.217	0.363	ns
		Hap2	CCGG	0.326	0.522	ns
		Hap3	CGGG	0.457	0.522	ns
	chr10_1317508	Hap1	CCCT	0.363	0.079	ns
		Hap2	CCTT	0.359	0.713	ns
		Hap3	CTTT	0.278	0.246	ns
	chr10_1571815	Hap1	CCCT	0.144	0.873	ns
		Hap2	CCTT	0.362	0.873	ns
		Hap3	CTTT	0.494	0.978	ns
	chr13_13467988	Hap1	CCCT	0.364	0.912	ns
		Hap2	CCTT	0.365	6.12 e^-04^	***
		Hap3	CTTT	0.270	0.001	***
	chr14_11301309	Hap1	AAAG	0.110	0.705	ns
		Hap2	AAGG	0.393	0.386	ns
		Hap3	AGGG	0.498	1.01 e^-20^	****
	chr14_11128124	Hap1	CCGG	0.414	6.59 e^-18^	****
	chr15_5858214	Hap1	CCCT	0.394	0.003	**
		Hap2	CCTT	0.192	0.242	ns
		Hap3	CTTT	0.315	0.057	ns
	chr17_15363223	Hap1	AAAT	0.539	1.20 e^-13^	****
Yield	chr17_19041958	Hap1	CCCT	0.146	0.516	ns
		Hap2	CCTT	0.281	0.516	ns
		Hap3	CTTT	0.370	0.799	ns
	chr19_9446619	Hap1	AAAG	0.349	0.002	**
		Hap2	AAGG	0.513	2.94 e^-06^	****
		Hap3	AGGG	0.059	0.872	ns
YMV	chr03_6338751	Hap1	GGGT	0.427	1.000	ns
		Hap2	GGTT	0.320	1.000	ns
		Hap3	GTTT	0.466	1.000	ns
	chr10_1116193	Hap1	AAAG	0.226	0.254	ns
		Hap2	AAGG	0.309	0.003	**
		Hap3	AGGG	0.465	6.75 e^-07^	****
	chr15_3906069	Hap1	AAAC	0.214	0.882	ns
		Hap2	AACC	0.281	0.882	ns
		Hap3	ACCC	0.412	0.882	ns
	chr16_1482029	Hap1	AAAT	0.307	0.096	ns
		Hap2	AATT	0.424	2.01 e^-04^	***
		Hap3	ATTT	0.576	0.006	**
	chr05_30671001	Hap1	AAAG	0.365	1.000	ns
		Hap2	AAGG	0.265	1.000	ns
		Hap3	AGGG	0.369	1.000	ns

ns=non-significant, *, **, ***, and **** indicate significant association between haplotypes and markers

.

## Discussion

### Phenotypic variation

The natural variation among the studied traits was high and very informative. Relatively high broad-sense heritability of 0.708 for tuber yield per plant and 0. 903 for yam mosaic virus severity score demonstrated substantial genetic variation in traits between the different clones. Therefore, the studied traits are amenable to genetic improvement through selection [40]. Furthermore, the observed natural genetic variation in the study materials signifies their relevance for genetic studies.

### Population differentiation

Understanding population structure within the studied clones is imperative to determine how it affects the ability of GWAS to infer marker-trait association. The population structure of the present study based on the delta reveals 7 sub-populations, indicating high genetic variability. The high genetic variability indicates the potentials of the studied clones for genetic improvement aimed at tuber yield per plant and yam mosaic virus. The the phylogeny analysis reveals similar results as the populature structure analysis, indicating their relevance in preventing sham associations in GWAS in this study [41, 42]. Thus, the marker density, diversity, and sample size demonstrated that the yam breeding panel used for this study is sufficiently powered to capture allelic variations for the studied traits.

### Genome-wide association studies

The whole-genome scan for phenotypic and allelic variation in tuber yield and yam mosaic virus resistance identified genome regions on ten chromosomes (chromosomes 4, 5, 8, 10, 13, 14, 15, 16, 17 and 19) with significant −log10 values. Both Q matrix (population structure) were considered in a mixed linear model for the association analysis to reduce false-positive associations. The model used for tuber yield and tolerance to yam mosaic virus showed no inflation of p-values indicating that the structure of relationships was well accounted for in the GWAS analysis. These findings are consistent with the view that traits with no inflation of p-values show that the structural relationship is adequate for GWAS analysis [42]. Genome-wide association mapping has been used in exploring the elite alleles of many agronomic traits such as tuber dry matter and oxidative browning [42] in water yam (*Dioscorea alata*). In the present study, the phenotypic effect values of the favorable alleles of TYP and YMV were evaluated and inferred to positively and negatively affect the individual traits. Based on the stringent criterion of −log10, we identified 17 significant markers trait associations ranging between 1.01 e-20 and 0.044 for tuber yield per plant; and 5 significant markers trait associations ranging between 5.25 e-14 and 0.029 for yam mosaic virus. The information on SNP variants from the present study would fast-track the application of genomics-informed selection decisions in breeding white Guinea yam for higher tuber yield and resistance to mosaic virus. Such great potential of GWAS has been reported for some root and tuber crops such as cassava [43], potatoes [44] and water yam [42].

### Detection of QTNs by multi-locus models (MLMs)

This study used different MLMs (FASTmrMLM, mrMLM, pKWmEB and pLARmEB) to identify genomic region associated with TYP and YMV. A total of 17 SNPs were significantly associated with TYP by the four MLM models across 9 out of the 20 chromosomes viz: chrs 4, 5, 8, 10, 14, 15, 17 and 19. Each of the four models detected different and complemeneted numbers of the SNPs: pKWmEB and pLARmEB (7 QTNs each) > FASTmrMLM, mrMLM (2 QTNs each). This indicates varied detection of each model. The MLMs used in this study detected putative candidate genes for the studied traits indicating its usefulness in GWAS. These results support the view that MLMs are useful for identifying QTNs and candidate genes in plants [45]. The findings of this study established a link between quantitative traits such as tuber yield and yam mosaic virus and single nucleotide polymorphisms. The variations observed in the population pannels constitute a pool of quantitative trait nucleotides (QTNs) that modulate tuber yield and yam mosaic virus traits in white yam.

### Identification of putative genes

Our results identified SNP markers that associate significantly with allelic variation for tuber yield and YMV tolerance in white yam. The detected markers offer good targets for further validation and analysis due to their location in proximity to candidate genes regulating growth, development and disease resistance. The SNP in chromosome 3 is near to AP2/ERF domain, AUX/IAA protein, major facilitator, sugar transporter-like genes. Zarei et al. [46] reported that the AP2/ERF-domain transcription factor ORA59 acts as the integrator of the jasmonic acid (JA) and ethylene (ET) signaling pathways and is the key regulator of JA- and ET-responsive PLANT DEFENSIN1.2 (PDF1.2) expression. The SNP in chromosome 4 is near to Geminivirus AL1 replication-associated protein, catalytic domain, AP2/ERF domain, NB-ARC, Dirigent protein, and membrane transport protein genes. The NB-ARC domain is noted to play a role in ATPase domain that comprises NB, ARC1, and ARC2 subdomains, which in its nucleotide-binding state regulates the R protein activity or resistance in plants [47]. The plant defense is induced by the R proteins in response to specific pathogen-derived molecules, called avirulence (AVR) proteins, thereby restricting pathogen proliferation [48]. The SNP in chromosome 10 is near to Geminivirus AL1 replication-associated protein, catalytic domain, Geminivirus Rep catalytic domain, Geminivirus AL3 coat protein, AP2/ERF domain, NB-ARC, Chlorophyll A-B binding protein, plant and chromista. Geminivirus AR1/BR1 coat protein, AP2/ERF domain, Geminivirus AL1 replication-associated protein, catalytic domain, Geminivirus AL1 replication-associated protein, central domain, and NB-ARC genes. Geminiviruses have been reported by Sunter and Bisaro [49] to play role in the Transactivation of Geminivirus AR1 and BR1 Gene Expression by the Viral AL2 Gene Product. Chlorophyll A-B binding protein is known as a light receptor that stimulates growth and development in plants [50]. The SNP in chromosome 16 is near to Geminivirus AR1/BR1 coat protein; AP2/ERF domain; Geminivirus AL1 replication-associated protein, catalytic domain; Geminivirus AL1 replication-associated protein, central domain; and NB-ARC genes. The SNP in chromosome 14 is near to expansin, cellulose-binding-like domain; mitochondrial substrate/solute carrier, expansin, root cap; dirigent protein; small auxin-up RNA; major facilitator, sugar transporter-like genes. Expansins or expansin-like proteins (loosenins) were reported to loosen plant cell wall activity and lignocellulose saccharification [51]. Mitochondrial carrier proteins play roles in plant growth and disease resistance [52]. The SNP in chromosome 15 is near to Gibberellin regulated protein; Major facilitator, sugar transporter-like; Senescence regulator S40; ABC transporter-like genes. The gibberellin regulated protein (GRP) has been noted to be up-regulated by gibberellin, and most of these proteins have a role in plant development and some of its members have antimicrobial activity [53, 54]. The SNP in chromosome 19 is near to Exportin-1/Importin-beta-like; Expansin; Sodium/calcium exchanger membrane region; Major facilitator, sugar transporter-like; AUX/IAA protein. The sodium/calcium exchanger has been reported to influence metabolic regulation on ion carrier interactions in living organisms [55]. The SNPs in chromosomes 6 and 8 are near to AUX/IAA protein and Protein ENHANCED DISEASE RESISTANCE 2, C-terminal (EDR2) genes. The Aux/IAA gene has been noted to play cellular and developmental roles in plants' lifespan, such as root development, shoot growth, and fruit ripening [56]. The Protein ENHANCED DISEASE RESISTANCE 2, C-terminal (EDR2) in plants limits cell death initiation and the establishment of hypersensitive response [57]. The identified putative candidate genes and SNPs linked with these important economic traits could help design new breeding strategies to hoard superior alleles for these key traits in future marker-based breeding. The novel regions identified in this study have not been previously detected, possibly due to the limitations of the various marker systems used in earlier studies.

Our findings indicated that multiple loci having unequal effects can influence the variation for TYP and YMV in white yam. The identified novel candidate genomic regions with growth, development and disease resistance genes in our study require further validation and testing in yam germplasm. This could be done by converting these MTAs into low cost Kompetitive Allele-Specific PCR (KASP) markers that can efficiently transfer alleles into elite yam genotypes as reported for wheat [58]. These valuable genomic resources and PCR based markers (KASP markers) could greatly support selection initiatives for key traits in yam breeding through marker-assisted selection (MAS). These will also support the systematic study of the genetics, comparative genomics and evolution of yam, aimed at expediting the isolation and characterization of genes that control agronomically important traits such as tuber yield and yam mosaic virus.

The SNP marker-TYP trait association exhibited high haplotype segregation. The marker effects alleles CC and CT are responsible for predicting high tuber yield per plant in the diversity panel used in the study, while alleles TT and GG were identified to associate with low yield. For the YMV, we found alleles GG, AG and TT to be responsible for low YMV disease scoring prediction. These findings suggest that data mining of favorable alleles is essential for improving the quantitative trait for tuber yield and YMV in yam using marker-assisted selection. Moreover, the results could be helpful for marker validation and deployment in yam breeding. Our findings agree with the view that information on marker effect based on segregation pattern is fundamental for marker validation and deployment in a breeding program [47, 59]. Association mapping has been utilized to explore elite alleles present in many agronomic traits, including yield and related attributes in bread wheat [60].

## Conclusion

Useful genetic variability exists in the 406 genotypes studied. The genetic architecture of TYP and YMV are regulated by varied QTNs unevenly distributed on the 20 chromosomes of white yam. Among the 4 MLM models, pKWmEB and pLARmEB are most robust in identifying more QTNs. The associated SNP markers could be potentially employed for targeted and accelerated tuber yield per plant and YMV resistance in white yam. The information from our study could help design new breeding strategies to hoard superior alleles for tuber yield per plant and yam mosaic virus in future marker-based breeding. The chromosomal regions controlling these studied traits could be exploited for selection and effective pyramiding of favorable alleles in white yam population improvement. Findings are relevant for population improvement of desirable TYP and YMV traits using marker assisted breeding (MAB) and haplotype-based scheme.

## Supplementary Information

**Additional file 1: Supplementary Table 1.** Description of trait progenitors utilized for the study. **Supplementary Table 2.** BLUP values of tuber yield per plant (TYP) and yam mosaic virus (YMV) among 406 clones of white yam. **Supplementary Table 3.** Cluster membership of 406 genotypes of white yam based on structure and phylogeny tree analyses. **Supplementary Table 4.** Single nucleotide polymorphism (SNP) markers associated with the yield per plant (TYP) and yam mosaic virus (YMV) and putative genes identified in chromosomes of 406 clones of white yam

## Data Availability

The Variant Call Format (VCF) file used in this study for the analysis can be viewed on www.yambase.org under genotypic data through this link https://yambase.org/breeders/trial/445?format= . Associated phenotypic data is presented as supplementary table within the document.
