# Supplementary material for: Identification of quantitative trait nucleotides and candidate genes for tuber yield and mosaic virus tolerance in an elite population of white guinea yam (Dioscorea rotundata) using genome-wide association scan
Source: BMC Plant Biol. 2021 Nov 22;21:552. doi: 10.1186/s12870-021-03314-w (PMC8607609; doi:10.1186/s12870-021-03314-w)
Supplement: Supplementary file 1 — Additional file 1: Supplementary Table 1. Description of trait progenitors utilized for the study. Supplementary Table 2. BLUP values of tuber yield per plant (TYP) and yam mosaic virus (YMV) among 406 clones of white yam. Supplementary Table 3. Cluster membership of 406 genotypes of white yam based on structure and phylogeny tree analyses. Supplementary Table 4. Single nucleotide polymorphism (SNP) markers associated with the yield per plant (TYP) and yam mosaic virus (YMV) and putative genes identified in chromosomes of 406 clones of white yam [file 12870_2021_3314_MOESM1_ESM.docx]

**Supplementary figure 1:** SNP distribution and density across chromosome alongside SNP mutation and SNP summary: A (SNP density and distribution); B (Transition/Transversion); C (distribution of expected heterozygosity); D (distribution of observed); E (distribution of minor allele frequency) and F (distribution of polymorphic information content).

Table S1. Description of trait progenitors utilized for the study

| **SN** | **Entry name** | **Status** | **Pedigree** |
| --- | --- | --- | --- |
| 1 | TDr1602_4 | Improved | TDr8902157/TDr9902789 |
| 2 | TDr1602_5 | Improved | TDr8902157/TDr9902789 |
| 3 | TDr1603_2 | Improved | TDr8902475/TDr9902789 |
| 4 | TDr1603_3 | Improved | TDr8902475/TDr9902789 |
| 5 | TDr1603_5 | Improved | TDr8902475/TDr9902789 |
| 6 | TDr1606_16 | Improved | TDr08-21-3/TDr99/02607 |
| 7 | TDr1606_6 | Improved | TDr08-21-3/TDr99/02607 |
| 8 | TDr1601_11 | Improved | TDr9700793/TDr9902789 |
| 9 | TDr1601_12 | Improved | TDr9700793/TDr9902789 |
| 10 | TDr1601_9 | Improved | TDr9700793/TDr9902789 |
| 11 | TDr1607_10 | Improved | TDr9519158/TDr9501932 |
| 12 | TDr1608_24 | Improved | TDr9518988/TDr9501932 |
| 13 | TDr1608_36 | Improved | TDr9518988/TDr9501932 |
| 14 | TDr1609_14 | Improved | TDrOju-iyawo/TDr9501932 |
| 15 | TDr1609_29 | Improved | TDrOju-iyawo/TDr9501932 |
| 16 | TDr1609_30 | Improved | TDrOju-iyawo/TDr9501932 |
| 17 | TDr1604_56 | Improved | TDr9700632/TDr9902607 |
| 18 | TDr1604_58 | Improved | TDr9700632/TDr9902607 |
| 19 | TDr1604_60 | Improved | TDr9700632/TDr9902607 |
| 20 | TDr8902157_1 | Improved | TDr8902157/TDr9902607 |
| 21 | TDr8902157_103 | Improved | TDr8902157/TDr9902607 |
| 22 | TDr8902157_16 | Improved | TDr8902157/TDr9902607 |
| 23 | TDr8902157_24 | Improved |  |
| 24 | TDr8902157_3 | Improved | TDr8902157/TDr9902607 |
| 25 | TDr8902157_37 | Improved | TDr8902157/TDr9902607 |
| 26 | TDr8902157_4 | Improved | TDr8902157/TDr9902607 |
| 27 | TDr8902157_48 | Improved | TDr8902157/TDr9902607 |
| 28 | TDr8902157_49 | Improved | TDr8902157/TDr9902607 |
| 29 | TDr8902157_55 | Improved | TDr8902157/TDr9902607 |
| 30 | TDr8902157_57 | Improved | TDr8902157/TDr9902607 |
| 31 | TDr8902157_58 | Improved | TDr8902157/TDr9902607 |
| 32 | TDr8902157_64 | Improved | TDr8902157/TDr9902607 |
| 33 | TDr8902157_66 | Improved | TDr8902157/TDr9902607 |
| 34 | TDr8902157_67 | Improved | TDr8902157/TDr9902607 |
| 35 | TDr8902157_69 | Improved | TDr8902157/TDr9902607 |
| 36 | TDr8902157_72 | Improved | TDr8902157/TDr9902607 |
| **37** | TDr8902157_73 | Improved | TDr8902157/TDr9902607 |
| 38 | TDr8902157_76 | Improved |  |
| 39 | TDr8902157_78 | Improved | TDr8902157/TDr9902607 |
| 40 | TDr8902157_79 | Improved | TDr8902157/TDr9902607 |
| 41 | TDr8902157_8 | Improved |  |
| 42 | TDr8902157_81 | Improved | TDr8902157/TDr9902607 |
| 43 | TDr8902157_87 | Improved | TDr8902157/TDr9902607 |
| 44 | TDr8902157_91 | Improved | TDr8902157/TDr9902607 |

Table S1. Continued. Description of trait progenitors utilized for the study

| **SN** | **Entry name** | **Status** | **Pedigree** |
| --- | --- | --- | --- |
| 45 | TDr8902157_94 | Improved | TDr8902157/TDr9902607 |
| 46 | TDr8902157_74 | Improved | TDr8902157/TDr9501932 |
| 47 | TDr8902157_17 | Improved |  |
| 48 | TDr8902157_2 | Improved | TDr8902157/TDr9501932 |
| 49 | TDr8902157_29 | Improved | TDr8902157/TDr9501932 |
| 50 | TDr8902157_30 | Improved | TDr8902157/TDr9501932 |
| 51 | TDr8902157_32 | Improved | TDr8902157/TDr9501932 |
| 52 | TDr8902157_43 | Improved |  |
| 53 | TDr8902157_45 | Improved |  |
| 54 | TDr8902157_62 | Improved | TDr8902157/TDr9501932 |
| 55 | TDr8902157_65 | Improved | TDr8902157/TDr9501932 |
| 56 | TDr8902157_71 | Improved | TDr8902157/TDr9501932 |
| 57 | TDr8902157_77 | Improved |  |
| 58 | TDr8902157_82 | Improved | TDr8902157/TDr9501932 |
| 59 | TDr8902157_84 | Improved | TDr8902157/TDr9501932 |
| 60 | TDr8902157_88 | Improved |  |
| 61 | TDr8902157_89 | Improved | TDr8902157/TDr9501932 |
| 62 | TDr8902157_96 | Improved |  |
| 63 | TDr8902475_113 | Improved | TDr8902475/TDr9501932 |
| 64 | TDr8902475_94 | Improved | TDr8902475/TDr9501932 |
| 65 | TDr8902475_99 | Improved | TDr8902475/TDr9501932 |
| 66 | TDr8902475_100 | Improved | TDr8902475/TDr9501932 |
| 67 | TDr8902475_102 | Improved | TDr8902475/TDr9501932 |
| 68 | TDr8902475_104 | Improved | TDr8902475/TDr9501932 |
| 69 | TDr8902475_106 | Improved | TDr8902475/TDr9501932 |
| 70 | TDr8902475_107 | Improved | TDr8902475/TDr9501932 |
| 71 | TDr8902475_109 | Improved | TDr8902475/TDr9501932 |
| 72 | TDr8902475_115 | Improved | TDr8902475/TDr9501932 |
| 73 | TDr8902475_118 | Improved | TDr8902475/TDr9501932 |
| 74 | TDr8902475_122 | Improved | TDr8902475/TDr9501932 |
| 75 | TDr8902475_127 | Improved | TDr8902475/TDr9501932 |
| 76 | TDr8902475_128 | Improved | TDr8902475/TDr9501932 |
| 77 | TDr8902475_130 | Improved | TDr8902475/TDr9501932 |
| 78 | TDr8902475_54 | Improved | TDr8902475/TDr9501932 |
| 79 | TDr8902475_63 | Improved | TDr8902475/TDr9501932 |
| 80 | TDr8902475_98 | Improved | TDr8902475/TDr9501932 |
| 81 | TDr9518988_44 | Improved | TDr9518988/TDr9902607 |
| 82 | TDr9518988_58 | Improved | TDr9518988/TDr9902607 |
| 83 | TDr9518988_62 | Improved | TDr9518988/TDr9902607 |
| 84 | TDr9518988_12 | Improved | TDr9518988/TDr9501932 |
| 85 | TDr9518988_14 | Improved | TDr9518988/TDr9501932 |
| 86 | TDr9518988_17 | Improved | TDr9518988/TDr9501932 |
| 87 | TDr9518988_20 | Improved | TDr9518988/TDr9501932 |
| 88 | TDr9518988_23 | Improved | TDr9518988/TDr9501932 |

Table S1. Continued. Description of trait progenitors utilized for the study

| **SN** | **Entry name** | **Status** | **Pedigree** |
| --- | --- | --- | --- |
| 89 | TDr9518988_24 | Improved | TDr9518988/TDr9501932 |
| 90 | TDr9518988_27 | Improved | TDr9518988/TDr9501932 |
| 91 | TDr9518988_28 | Improved | TDr9518988/TDr9501932 |
| 92 | TDr9518988_30 | Improved | TDr9518988/TDr9501932 |
| 93 | TDr9518988_34 | Improved | TDr9518988/TDr9501932 |
| 94 | TDr9518988_35 | Improved | TDr9518988/TDr9501932 |
| 95 | TDr9518988_36 | Improved | TDr9518988/TDr9501932 |
| 96 | TDr9518988_39 | Improved | TDr9518988/TDr9501932 |
| 97 | TDr9518988_4 | Improved | TDr9518988/TDr9501932 |
| 98 | TDr9518988_40 | Improved | TDr9518988/TDr9501932 |
| 99 | TDr9518988_41 | Improved | TDr9518988/TDr9501932 |
| 100 | TDr9518988_42 | Improved | TDr9518988/TDr9501932 |
| 101 | TDr9518988_46 | Improved | TDr9518988/TDr9501932 |
| 102 | TDr9518988_48 | Improved | TDr9518988/TDr9501932 |
| 103 | TDr9518988_50 | Improved | TDr9518988/TDr9501932 |
| 104 | TDr9518988_51 | Improved | TDr9518988/TDr9501932 |
| 105 | TDr9518988_52 | Improved | TDr9518988/TDr9501932 |
| 106 | TDr9518988_55 | Improved | TDr9518988/TDr9501932 |
| 107 | TDr9518988_59 | Improved | TDr9518988/TDr9501932 |
| 108 | TDr9518988_6 | Improved | TDr9518988/TDr9501932 |
| 109 | TDr9518988_60 | Improved | TDr9518988/TDr9501932 |
| 110 | TDr9518988_63 | Improved | TDr9518988/TDr9501932 |
| 111 | TDr9518988_64 | Improved | TDr9518988/TDr9501932 |
| 112 | TDr9518988_7 | Improved | TDr9518988/TDr9501932 |
| 113 | TDr9518988_70 | Improved | TDr9518988/TDr9501932 |
| 114 | TDr9518988_71 | Improved | TDr9518988/TDr9501932 |
| 115 | TDr9518988_72 | Improved | TDr9518988/TDr9501932 |
| 116 | TDr9518988_73 | Improved | TDr9518988/TDr9501932 |
| 117 | TDr9518988_74 | Improved | TDr9518988/TDr9501932 |
| 118 | TDr9518988_76 | Improved | TDr9518988/TDr9501932 |
| 119 | TDr9518988_77 | Improved | TDr9518988/TDr9501932 |
| 120 | TDr9518988_78 | Improved | TDr9518988/TDr9501932 |
| 121 | TDr9518988_79 | Improved | TDr9518988/TDr9501932 |
| **122** | TDr9518988_8 | Improved | TDr9518988/TDr9501932 |
| 123 | TDr9518988_80 | Improved | TDr9518988/TDr9501932 |
| 124 | TDr9518988_81 | Improved | TDr9518988/TDr9501932 |
| 125 | TDr9518988_82 | Improved | TDr9518988/TDr9501932 |
| 126 | TDr9518988_83 | Improved | TDr9518988/TDr9501932 |
| 127 | TDr9518988_9 | Improved | TDr9518988/TDr9501932 |
| 128 | TDr9519158_12 | Improved | TDr9519158/TDr9902607 |
| 129 | TDr9519158_56 | Improved | TDr9519158/TDr9902607 |
| 130 | TDr9519158_11 | Improved | TDr9519158/TDr9501932 |
| 131 | TDr9519158_13 | Improved | TDr9519158/TDr9501932 |
| 132 | TDr9519158_14 | Improved | TDr9519158/TDr9501932 |

Table S1. Continued. Description of trait progenitors utilized for the study

| **SN** | **Entry name** | **Status** | **Pedigree** |
| --- | --- | --- | --- |
| 133 | TDr9519158_16 | Improved | TDr9519158/TDr9501932 |
| 134 | TDr9519158_17 | Improved | TDr9519158/TDr9501932 |
| 135 | TDr9519158_18 | Improved | TDr9519158/TDr9501932 |
| 136 | TDr9519158_19 | Improved | TDr9519158/TDr9501932 |
| 137 | TDr9519158_2 | Improved | TDr9519158/TDr9501932 |
| 138 | TDr9519158_23 | Improved | TDr9519158/TDr9501932 |
| 139 | TDr9519158_24 | Improved | TDr9519158/TDr9501932 |
| 140 | TDr9519158_27 | Improved | TDr9519158/TDr9501932 |
| 141 | TDr9519158_28 | Improved | TDr9519158/TDr9501932 |
| 142 | TDr9519158_3 | Improved | TDr9519158/TDr9501932 |
| 143 | TDr9519158_30 | Improved | TDr9519158/TDr9501932 |
| 144 | TDr9519158_31 | Improved | TDr9519158/TDr9501932 |
| 145 | TDr9519158_33 | Improved | TDr9519158/TDr9501932 |
| 146 | TDr9519158_34 | Improved | TDr9519158/TDr9501932 |
| 147 | TDr9519158_35 | Improved | TDr9519158/TDr9501932 |
| 148 | TDr9519158_36 | Improved | TDr9519158/TDr9501932 |
| 149 | TDr9519158_38 | Improved | TDr9519158/TDr9501932 |
| 150 | TDr9519158_39 | Improved | TDr9519158/TDr9501932 |
| 151 | TDr9519158_4 | Improved | TDr9519158/TDr9501932 |
| 152 | TDr9519158_40 | Improved | TDr9519158/TDr9501932 |
| 153 | TDr9519158_41 | Improved | TDr9519158/TDr9501932 |
| 154 | TDr9519158_42 | Improved | TDr9519158/TDr9501932 |
| 155 | TDr9519158_43 | Improved | TDr9519158/TDr9501932 |
| 156 | TDr9519158_46 | Improved | TDr9519158/TDr9501932 |
| 157 | TDr9519158_48 | Improved | TDr9519158/TDr9501932 |
| 158 | TDr9519158_50 | Improved | TDr9519158/TDr9501932 |
| 159 | TDr9519158_51 | Improved | TDr9519158/TDr9501932 |
| 160 | TDr9519158_52 | Improved | TDr9519158/TDr9501932 |
| 161 | TDr9519158_53 | Improved | TDr9519158/TDr9501932 |
| 162 | TDr9519158_55 | Improved | TDr9519158/TDr9501932 |
| 163 | TDr9519158_7 | Improved | TDr9519158/TDr9501932 |
| 164 | TDrOjuiyawo_108 | Improved | TDrOjuiyawo/TDr9902607 |
| 165 | TDrOjuiyawo_113 | Improved | TDrOjuiyawo/TDr9902607 |
| 166 | TDrOjuiyawo_146 | Improved | TDrOjuiyawo/TDr9902607 |
| 167 | TDrOjuiyawo_155 | Improved | TDrOjuiyawo/TDr9902607 |
| 168 | TDrOjuiyawo_159 | Improved | TDrOjuiyawo/TDr9902607 |
| 169 | TDrOjuiyawo_160 | Improved | TDrOjuiyawo/TDr9902607 |
| 170 | TDrOjuiyawo_169 | Improved | TDrOjuiyawo/TDr9902607 |
| 171 | TDrOjuiyawo_171 | Improved | TDrOjuiyawo/TDr9902607 |
| 172 | TDrOjuiyawo_173 | Improved | TDrOjuiyawo/TDr9902607 |
| 173 | TDrOjuiyawo_35 | Improved | TDrOjuiyawo/TDr9902607 |
| 174 | TDrOjuiyawo_39 | Improved | TDrOjuiyawo/TDr9902607 |
| 175 | TDrOjuiyawo_53 | Improved | TDrOjuiyawo/TDr9902607 |
| 176 | TDrOjuiyawo_54 | Improved | TDrOjuiyawo/TDr9902607 |

Table S1. Description of trait progenitors utilized for the study

| **SN** | **Entry name** | **Status** | **Pedigree** |
| --- | --- | --- | --- |
| 177 | TDrOjuiyawo_60 | Improved | TDrOjuiyawo/TDr9902607 |
| 178 | TDrOjuiyawo_61 | Improved | TDrOjuiyawo/TDr9902607 |
| 179 | TDrOjuiyawo_67 | Improved | TDrOjuiyawo/TDr9902607 |
| 180 | TDrOjuiyawo_71 | Improved | TDrOjuiyawo/TDr9902607 |
| 181 | TDrOjuiyawo_75 | Improved | TDrOjuiyawo/TDr9902607 |
| 182 | TDrOjuiyawo_81 | Improved | TDrOjuiyawo/TDr9902607 |
| 183 | TDrOjuiyawo_82 | Improved | TDrOjuiyawo/TDr9902607 |
| 184 | TDrOjuiyawo_85 | Improved | TDrOjuiyawo/TDr9902607 |
| 185 | TDrOjuiyawo_88 | Improved | TDrOjuiyawo/TDr9902607 |
| 186 | TDrOjuiyawo_89 | Improved | TDrOjuiyawo/TDr9902607 |
| 187 | TDrOjuiyawo_96 | Improved | TDrOjuiyawo/TDr9902607 |
| 188 | TDrOjuiyawo_97 | Improved | TDrOjuiyawo/TDr9902607 |
| 189 | TDrOjuiyawo_116 | Improved |  |
| 190 | TDrOjuiyawo_129 | Improved | TDrOjuiyawo/TDr9501932 |
| 191 | TDrOjuiyawo_132 | Improved | TDrOjuiyawo/TDr9501932 |
| 192 | TDrOjuiyawo_143 | Improved | TDrOjuiyawo/TDr9501932 |
| 193 | TDrOjuiyawo_145 | Improved | TDrOjuiyawo/TDr9501932 |
| 194 | TDrOjuiyawo_150 | Improved | TDrOjuiyawo/TDr9501932 |
| 195 | TDrOjuiyawo_157 | Improved | TDrOjuiyawo/TDr9501932 |
| 196 | TDrOjuiyawo_162 | Improved | TDrOjuiyawo/TDr9501932 |
| 197 | TDrOjuiyawo_163 | Improved | TDrOjuiyawo/TDr9501932 |
| 198 | TDrOjuiyawo_167 | Improved | TDrOjuiyawo/TDr9501932 |
| 199 | TDrOjuiyawo_17 | Improved | TDrOjuiyawo/TDr9501932 |
| 200 | TDrOjuiyawo_176 | Improved | TDrOjuiyawo/TDr9501932 |
| 201 | TDrOjuiyawo_177 | Improved | TDrOjuiyawo/TDr9501932 |
| 202 | TDrOjuiyawo_185 | Improved | TDrOjuiyawo/TDr9501932 |
| 203 | TDrOjuiyawo_19 | Improved | TDrOjuiyawo/TDr9501932 |
| 204 | TDrOjuiyawo_33 | Improved | TDrOjuiyawo/TDr9501932 |
| 205 | TDrOjuiyawo_52 | Improved | TDrOjuiyawo/TDr9501932 |
| 206 | TDrOjuiyawo_56 | Improved | TDrOjuiyawo/TDr9501932 |
| 207 | TDrOjuiyawo_64 | Improved | TDrOjuiyawo/TDr9501932 |
| 208 | TDrOjuiyawo_91 | Improved | TDrOjuiyawo/TDr9501932 |
| 209 | TDr9700793_1 | Improved | TDr9700793/TDr9501932 |
| 210 | TDr9700793_101 | Improved | TDr9700793/TDr9501932 |
| 211 | TDr9700793_102 | Improved | TDr9700793/TDr9501932 |
| 212 | TDr9700793_106 | Improved | TDr9700793/TDr9902607 |
| 213 | TDr9700793_107 | Improved | TDr9700793/TDr9902607 |
| 214 | TDr9700793_11 | Improved | TDr9700793/TDr9902607 |
| 215 | TDr9700793_123 | Improved | TDr9700793/TDr9501932 |
| 216 | TDr9700793_124 | Improved | TDr9700793/TDr9501932 |
| 217 | TDr9700793_125 | Improved | TDr9700793/TDr9902607 |
| 218 | TDr9700793_127 | Improved | TDr9700793/TDr9501932 |
| 219 | TDr9700793_13 | Improved | TDr9700793/TDr9902607 |
| 220 | TDr9700793_133 | Improved | TDr9700793/TDr9501932 |

Table S1. Continued. Description of trait progenitors utilized for the study

| **SN** | **Entry name** | **Status** | **Pedigree** |
| --- | --- | --- | --- |
| 221 | TDr9700793_136 | Improved | TDr9700793/TDr9902607 |
| 222 | TDr9700793_137 | Improved | TDr9700793/TDr9501932 |
| 223 | TDr9700793_138 | Improved | TDr9700793/TDr9501932 |
| 224 | TDr9700793_139 | Improved | TDr9700793/TDr9902607 |
| 225 | TDr9700793_143 | Improved | TDr9700793/TDr9902607 |
| 226 | TDr9700793_145 | Improved | TDr9700793/TDr9902607 |
| 227 | TDr9700793_146 | Improved | TDr9700793/TDr9902607 |
| 228 | TDr9700793_147 | Improved | TDr9700793/TDr9501932 |
| 229 | TDr9700793_148 | Improved | TDr9700793/TDr9902607 |
| 230 | TDr9700793_15 | Improved | TDr9700793/TDr9501932 |
| 231 | TDr9700793_151 | Improved | TDr9700793/TDr9501932 |
| 232 | TDr9700793_155 | Improved | TDr9700793/TDr9501932 |
| 233 | TDr9700793_156 | Improved | TDr9700793/TDr9501932 |
| 234 | TDr9700793_159 | Improved | TDr9700793/TDr9501932 |
| 235 | TDr9700793_16 | Improved | TDr9700793/TDr9501932 |
| 236 | TDr9700793_160 | Improved | TDr9700793/TDr9902607 |
| 237 | TDr9700793_163 | Improved | TDr9700793/TDr9902607 |
| 238 | TDr9700793_18 | Improved | TDr9700793/TDr9501932 |
| 239 | TDr9700793_20 | Improved | TDr9700793/TDr9501932 |
| 240 | TDr9700793_21 | Improved | TDr9700793/TDr9902607 |
| 241 | TDr9700793_22 | Improved | TDr9700793/TDr9902607 |
| 242 | TDr9700793_23 | Improved | TDr9700793/TDr9902607 |
| 243 | TDr9700793_24 | Improved | TDr9700793/TDr9902607 |
| 244 | TDr9700793_25 | Improved | TDr9700793/TDr9902789 |
| 245 | TDr9700793_26 | Improved | TDr9700793/TDr9501932 |
| 246 | TDr9700793_29 | Improved | TDr9700793/TDr9501932 |
| 247 | TDr9700793_33 | Improved | TDr9700793/TDr9501932 |
| 248 | TDr9700793_35 | Improved | TDr9700793/TDr9902789 |
| 249 | TDr9700793_36 | Improved | TDr9700793/TDr9501932 |
| 250 | TDr9700793_37 | Improved | TDr9700793/TDr9501932 |
| 251 | TDr9700793_38 | Improved | TDr9700793/TDr9501932 |
| 252 | TDr9700793_5 | Improved | TDr9700205/TDr9501932 |
| 253 | TDr9700793_51 | Improved | TDr9700205/TDr9902607 |
| 254 | TDr9700793_71 | Improved | TDr9700205/TDr9501932 |
| 255 | TDr9700793_8 | Improved | TDr9700205/TDr9902607 |
| 256 | TDr9700205_104 | Improved | TDr9700205/TDr9501932 |
| 257 | TDr9700205_106 | Improved | TDr9700205/TDr9902607 |
| 258 | TDr9700205_111 | Improved | TDr9700205/TDr9501932 |
| 259 | TDr9700205_112 | Improved | TDr9700205/TDr9501932 |
| 260 | TDr9700205_150 | Improved | TDr9700205/TDr9902789 |
| 261 | TDr9700205_153 | Improved | TDr9700205/TDr9501932 |
| 262 | TDr9700205_158 | Improved | TDr9700205/TDr9501932 |
| 263 | TDr9700205_177 | Improved | TDr9700205/TDr9501932 |
| 264 | TDr9700205_181 | Improved | TDr9700205/TDr9501932 |

Table S1. Continued. Description of trait progenitors utilized for the study

| **SN** | **Entry name** | **Status** | **Pedigree** |
| --- | --- | --- | --- |
| 265 | TDr9700205_182 | Improved |  |
| 266 | TDr9700205_186 | Improved | TDr9700205/TDr9501932 |
| 267 | TDr9700205_196 | Improved |  |
| 268 | TDr9700205_209 | Improved |  |
| 269 | TDr9700205_211 | Improved |  |
| 270 | TDr9700205_216 | Improved |  |
| 271 | TDr9700205_223 | Improved | TDr9700205/TDr9902607 |
| 272 | TDr9700205_225 | Improved | TDr9700205/TDr9501932 |
| 273 | TDr9700205_227 | Improved | TDr9700205/TDr9902607 |
| 274 | TDr9700205_231 | Improved | TDr9700205/TDr9902607 |
| 275 | TDr9700205_234 | Improved | TDr9700205/TDr9902789 |
| 276 | TDr9700205_238 | Improved | TDr9700205/TDr9902789 |
| 277 | TDr9700205_24 | Improved |  |
| 278 | TDr9700205_247 | Improved | TDr9700205/TDr9501932 |
| 279 | TDr9700205_249 | Improved |  |
| 280 | TDr9700205_253 | Improved | TDr9700205/TDr9501932 |
| 281 | TDr9700205_256 | Improved | TDr9700205/TDr9501932 |
| 282 | TDr9700205_257 | Improved | TDr9700205/TDr9902789 |
| 283 | TDr9700205_259 | Improved | TDr9700205/TDr9501932 |
| 284 | TDr9700205_28 | Improved |  |
| 285 | TDr9700205_30 | Improved |  |
| 286 | TDr9700205_31 | Improved |  |
| 287 | TDr9700205_32 | Improved | TDr9700205/TDr9902789 |
| 288 | TDr9700205_42 | Improved | TDr9700205/TDr9501932 |
| 289 | TDr9700632_11 | Improved | TDr9700632/TDr9501932 |
| 290 | TDr9700632_12 | Improved | TDr9700632/TDr9501932 |
| 291 | TDr9700632_16 | Improved | TDr9700632/TDr9501932 |
| 292 | TDr9700632_2 | Improved | TDr9700632/TDr9902607 |
| 293 | TDr9700632_21 | Improved | TDr9700632/TDr9902607 |
| 294 | TDr9700632_23 | Improved | TDr9700632/TDr9501932 |
| 295 | TDr9700632_25 | Improved | TDr9700632/TDr9902607 |
| 296 | TDr9700632_29 | Improved | TDr9700632/TDr9501932 |
| 297 | TDr9700632_3 | Improved | TDr9700632/TDr9902607 |
| 298 | TDr9700632_30 | Improved | TDr9700632/TDr9902607 |
| 299 | TDr9700632_31 | Improved | TDr9700632/TDr9501932 |
| 300 | TDr9700632_33 | Improved | TDr9700632/TDr9501932 |
| 301 | TDr9700632_34 | Improved | TDr9700632/TDr9501932 |
| 302 | TDr9700632_35 | Improved | TDr9700632/TDr9501932 |
| 303 | TDr9700632_36 | Improved | TDr9700632/TDr9501932 |
| 304 | TDr9700632_4 | Improved | TDr9700632/TDr9902607 |
| 305 | TDr9700632_41 | Improved | TDr9700632/TDr9902607 |
| 306 | TDr9700632_42 | Improved | TDr9700632/TDr9501932 |
| 307 | TDr9700632_44 | Improved | TDr9700632/TDr9902607 |
| 308 | TDr9700632_45 | Improved | TDr9700632/TDr9501932 |

Table S1. Continued. Description of trait progenitors utilized for the study

| **SN** | **Entry name** | **Status** | **Pedigree** |
| --- | --- | --- | --- |
| 309 | TDr9700632_48 | Improved | TDr9700632/TDr9902607 |
| 310 | TDr9700632_51 | Improved | TDr9700632/TDr9902607 |
| 311 | TDr9700632_53 | Improved | TDr9700632/TDr9902607 |
| 312 | TDr9700632_54 | Improved | TDr9700632/TDr9501932 |
| 313 | TDr9700632_58 | Improved | TDr9700632/TDr9902607 |
| 314 | TDr9700632_59 | Improved | TDr9700632/TDr9902607 |
| 315 | TDr9700632_6 | Improved | TDr9700632/TDr9501932 |
| 316 | TDr9700632_60 | Improved | TDr9700632/TDr9902607 |
| 317 | TDr9700632_63 | Improved | TDr9700632/TDr9902607 |
| 318 | TDr9700632_67 | Improved | TDr9700632/TDr9902607 |
| 319 | TDr9700632_69 | Improved | TDr9700632/TDr9902607 |
| 320 | TDr9700632_70 | Improved | TDr9700632/TDr9501932 |
| 321 | TDr9700632_72 | Improved | TDr9700632/TDr9902607 |
| 322 | TDr9700632_77 | Improved | TDr9700632/TDr9902607 |
| 323 | TDr9700632_8 | Improved | TDr9700632/TDr9501932 |
| 324 | TDr9700632_83 | Improved | TDr9700632/TDr9902607 |
| 325 | TDr9700632_84 | Improved | TDr9700632/TDr9902607 |
| 326 | TDr9700632_86 | Improved | TDr9700632/TDr9902607 |
| 327 | TDr9700632_87 | Improved | TDr9700632/TDr9501932 |
| 328 | TDr0000001 | Improved | TDr9700793/TDr9501932 |
| 329 | TDr0000362 | Improved | Unknown |
| 330 | TDr0900052 | Improved | TDr9700793/TDr9501932 |
| 331 | TDr0900121 | Improved | TDr9700793/TDr9501932 |
| 332 | TDr0900122 | Improved | TDr9700793/TDr9501932 |
| 333 | TDr0900134 | Improved | TDr9700793/TDr9501932 |
| 334 | TDr0900135 | Improved | TDr9700793/TDr9501932 |
| 335 | TDr0900152 | Improved | TDr9700793/TDr9501932 |
| 336 | TDr0900220 | Improved | TDr9700793/TDr9501932 |
| 337 | TDr0900263 | Improved | TDr9700793/TDr9501932 |
| 338 | TDr0900267 | Improved | TDr9700793/TDr9501932 |
| 339 | TDr0900295 | Improved | TDr9700793/TDr9501932 |
| 340 | TDr0900341 | Improved | TDr97/00793/TDr9501932 |
| 341 | TDr0900404 | Improved | TDr0701553/TDr9501932 |
| 342 | TDr0900408 | Improved | TDr0701553/TDr9501932 |
| 343 | TDr0902079 | Improved | TDr0701553 (OP) |
| 344 | TDr1000021 | Improved | TDr9518544/TDr9501932 |
| 345 | TDr1000052 | Improved | TDr9518544/TDr9501932 |
| 346 | TDr1000060 | Improved | TDr9518544/TDr95/01932 |
| 347 | TDr1000144 | Improved | TDr9518544/TDr9501932 |
| 348 | TDr1000149 | Improved | TDr9518544/TDr9501932 |
| 349 | TDr1000228 | Improved | TDr9518544/TDr9501932 |
| 350 | TDr1000245 | Improved | TDr9518544/TDr9501932 |
| 351 | TDr1000248 | Improved | TDr9518544/TDr9501932 |
| 352 | TDr1000282 | Improved | TDr9518544/TDr9501932 |

Table S1. Continued. Description of trait progenitors utilized for the study

| **SN** | **Entry name** | **Status** | **Pedigree** |
| --- | --- | --- | --- |
| 353 | TDr1000310 | Improved | TDr9518544/TDr9501932 |
| 354 | TDr1000412 | Improved | TDr9518544/TDr9501932 |
| 355 | TDr1000563 | Improved | TDr9518544/TDr9501932 |
| 356 | TDr1000600 | Improved | TDr9518544/TDr9501932 |
| 357 | TDr1000605 | Improved | TDr9518544/TDr9501932 |
| 358 | TDr1001012 | Improved | TDr9518544/TDr9501932 |
| 359 | TDr1100008 | Improved | TDr04-219/TDr0000196 |
| 360 | TDr1100015 | Improved | TDr04-219/TDr0000196 |
| 361 | TDr1100055 | Improved | TDr04-219/TDr0000196 |
| 362 | TDr1100128 | Improved | TDr9700840/TDr9902626 |
| 363 | TDr1100180 | Improved | TDr9700840/TDr9902626 |
| 364 | TDr1100228 | Improved | TDr9700205/TDr9902626 |
| 365 | TDr1100291 | Improved | TDr9700205/TDr99/02626 |
| 366 | TDr1100629 | Improved | TDr9518544/Pouna |
| 367 | TDr1100734 | Improved | TDr06-3/TDr1892 |
| 368 | TDr1101142 | Improved | TDr9519158 (OP) |
| 369 | TDr1101272 | Improved | TDr9600604 (OP) |
| 370 | TDr1101408 | Improved | TDr9600604 (OP) |
| 371 | TDr1101701 | Improved | Agbanwobe (OP) |
| 372 | TDr8900983 | Improved | TDr9700917/TDr9902626 |
| 373 | TDr0900013 | Improved | TDr9700793/TDr9501932 |
| 374 | TDr04_219 | Landrace | Unknown |
| 375 | TDr06_15 | Landrace | Unknown |
| 376 | TDr0821_2 | Landrace | Unknown |
| 377 | TDr8902475 | Improved | Unknown |
| 378 | TDr8902157 | Improved | Unknown |
| 379 | TDr8902665 | Improved | Unknown |
| 380 | TDr8902677 | Improved | Unknown |
| 381 | TDr9501932 | Improved | TDr8600309/Unknown |
| 382 | TDr9518544 | Improved | TDr8700571/Unknown |
| 383 | TDr9518988 | Improved | TDr8700571/Unknown |
| 384 | TDr9519156 | Improved | Unknown |
| 385 | TDr9519158 | Improved | Unknown |
| 386 | TDr9519177 | Improved | Unknown |
| 387 | TDr9600629 | Improved | TDr93:1/Unknown |
| 388 | TDr9700205 | Improved | TDr8700839/TDr8700552 |
| 389 | TDr9700632 | Improved | TDr93:24/TDr8902494 |
| 390 | TDr9700917 | Improved | TDr8901892/IN94R-2 |
| 391 | TDr9902562 | Improved | TDr93:1/TDr9500858 |
| 392 | TDr9902626 | Improved | TDr93:25/TDr9500858 |
| 393 | TDr9902789 | Improved | TDr93:50/TDr9502026 (TDr9100194/Unknown) |
| 394 | TDrAgbanwobe | Local | Unknown |
| 395 | TDrAlumaco | Local | Unknown |
| 396 | TDrDanacha | Local | Unknown |

Table S1. Continued. Description of trait progenitors utilized for the study

| **SN** | **Entry name** | **Status** | **Pedigree** |
| --- | --- | --- | --- |
| 397 | TDrEhobia | Local | Unknown |
| 398 | TDrFakesta | Local | Unknown |
| 399 | TDrLeusi | Local | Unknown |
| 400 | TDrMeccakusa | Local | Unknown |
| 401 | TDrNndu | Local | Unknown |
| 402 | TDrOjuiyawo | Local | Unknown |
| 403 | TDrPampers | Local | Unknown |
| 404 | TDrPouna | Local | Unknown |
| 405 | TDrUfenyi | Local | Unknown |
| 406 | TDrYangbedu | Local | Unknown |

Table S2. Best linear unbiased predictor (BLUP) values of tuber yield per plant (TYP) and yam mosaic virus (YMV) among 406 clones of white yam

| Genotype | TYP | YMV | Genotype | TYP | YMV |
| --- | --- | --- | --- | --- | --- |
| TDr0000001 | 1.16 | 787.87 | TDr1100734 | 1.00 | 807.98 |
| TDr0000362 | 0.96 | 1425.02 | TDr1101142 | 1.04 | 837.88 |
| TDr04_219 | 0.93 | 1173.57 | TDr1101272 | 1.14 | 791.20 |
| TDr06_15 | 0.94 | 1070.04 | TDr1101408 | 1.18 | 746.45 |
| TDr0821_2 | 0.93 | 100.56 | TDr1101701 | 1.08 | 791.97 |
| TDr0900013 | 1.22 | 814.68 | TDr1601_11 | 1.08 | 640.11 |
| TDr0900052 | 1.22 | 760.93 | TDr1601_12 | 1.01 | 692.11 |
| TDr0900121 | 1.05 | 822.41 | TDr1601_9 | 0.96 | 670.95 |
| TDr0900122 | 1.18 | 780.14 | TDr1602_4 | 1.14 | 1568.91 |
| TDr0900134 | 0.98 | 754.21 | TDr1602_5 | 1.17 | 1250.52 |
| TDr0900135 | 1.20 | 743.50 | TDr1603_2 | 1.20 | 702.33 |
| TDr0900152 | 1.05 | 812.13 | TDr1603_3 | 1.15 | 2156.63 |
| TDr0900220 | 1.12 | 792.34 | TDr1603_5 | 1.20 | 689.58 |
| TDr0900263 | 1.11 | 787.69 | TDr1604_56 | 1.06 | 162.96 |
| TDr0900267 | 1.12 | 842.87 | TDr1604_58 | 1.06 | 123.08 |
| TDr0900295 | 1.15 | 740.27 | TDr1604_60 | 1.06 | 660.70 |
| TDr0900341 | 1.00 | 764.13 | TDr1606_16 | 1.10 | 1228.82 |
| TDr0900404 | 1.07 | 756.85 | TDr1606_6 | 1.00 | 684.76 |
| TDr0900408 | 1.25 | 794.20 | TDr1607_10 | 1.27 | 650.90 |
| TDr0902079 | 1.11 | 852.15 | TDr1608_24 | 1.22 | 1437.24 |
| TDr1000021 | 1.29 | 779.75 | TDr1608_36 | 1.31 | 652.95 |
| TDr1000052 | 1.21 | 883.30 | TDr1609_14 | 1.24 | 714.16 |
| TDr1000060 | 1.06 | 928.77 | TDr1609_29 | 1.26 | 683.26 |
| TDr1000144 | 1.16 | 817.88 | TDr1609_30 | 1.28 | 1246.65 |
| TDr1000149 | 1.13 | 834.16 | TDr8900983 | 1.16 | 708.89 |
| TDr1000228 | 0.97 | 875.56 | TDr8902157 | 1.28 | 952.07 |
| TDr1000245 | 1.19 | 748.74 | TDr8902157_1 | 1.16 | 115.29 |
| TDr1000248 | 1.14 | 947.18 | TDr8902157_103 | 1.14 | 739.68 |
| TDr1000282 | 1.16 | 979.26 | TDr8902157_16 | 1.18 | 1283.62 |
| TDr1000310 | 1.22 | 907.29 | TDr8902157_17 | 1.29 | 695.34 |
| TDr1000412 | 1.01 | 805.00 | TDr8902157_2 | 1.29 | 1261.38 |
| TDr1000563 | 1.10 | 803.16 | TDr8902157_24 | 1.25 | 503.57 |
| TDr1000600 | 1.13 | 731.60 | TDr8902157_29 | 1.31 | 488.75 |
| TDr1000605 | 1.24 | 737.42 | TDr8902157_3 | 1.13 | 2106.09 |
| TDr1001012 | 1.13 | 871.49 | TDr8902157_30 | 1.35 | 509.30 |
| TDr1100008 | 1.09 | 866.21 | TDr8902157_32 | 1.23 | 687.25 |
| TDr1100015 | 1.11 | 844.38 | TDr8902157_37 | 1.16 | 1251.62 |
| TDr1100055 | 1.17 | 738.15 | TDr8902157_4 | 1.06 | 2115.83 |
| TDr1100128 | 1.11 | 765.18 | TDr8902157_43 | 1.24 | 678.33 |
| TDr1100180 | 1.22 | 727.30 | TDr8902157_45 | 1.18 | 673.25 |
| TDr1100228 | 1.08 | 723.56 | TDr8902157_48 | 1.20 | 542.73 |
| TDr1100291 | 1.01 | 833.72 | TDr8902157_49 | 1.12 | 1485.10 |
| TDr1100629 | 1.28 | 880.90 | TDr8902157_55 | 1.13 | 692.96 |

Table S2. continued

| Genotype | TYP | YMV | Genotype | TYP | YMV |
| --- | --- | --- | --- | --- | --- |
| TDr8902157_57 | 1.17 | 564.63 | TDr8902475_98 | 1.24 | 711.51 |
| TDr8902157_58 | 1.25 | 1273.55 | TDr8902475_99 | 1.09 | 1265.35 |
| TDr8902157_62 | 1.36 | 1231.48 | TDr8902665 | 1.10 | 888.18 |
| TDr8902157_64 | 1.16 | 2119.80 | TDr8902677 | 0.98 | 1252.39 |
| TDr8902157_65 | 1.24 | 1255.13 | TDr9501932 | 1.35 | 849.13 |
| TDr8902157_66 | 1.18 | 2113.54 | TDr9518544 | 1.37 | 904.73 |
| TDr8902157_67 | 1.13 | 1849.99 | TDr9518988 | 1.24 | 818.14 |
| TDr8902157_69 | 1.20 | 2101.98 | TDr9518988_12 | 1.33 | 1288.36 |
| TDr8902157_71 | 1.21 | 670.93 | TDr9518988_14 | 1.37 | 1261.08 |
| TDr8902157_72 | 1.20 | 124.17 | TDr9518988_17 | 1.28 | 744.03 |
| TDr8902157_73 | 1.18 | 1546.30 | TDr9518988_20 | 1.38 | 1286.46 |
| TDr8902157_74 | 1.06 | 1243.97 | TDr9518988_23 | 1.35 | 706.74 |
| TDr8902157_76 | 1.25 | 508.51 | TDr9518988_24 | 1.36 | 776.22 |
| TDr8902157_77 | 1.39 | 1510.75 | TDr9518988_27 | 1.23 | 739.83 |
| TDr8902157_78 | 1.05 | 1579.15 | TDr9518988_28 | 1.33 | 735.35 |
| TDr8902157_79 | 1.08 | 2101.70 | TDr9518988_30 | 1.38 | 742.13 |
| TDr8902157_8 | 1.12 | 182.82 | TDr9518988_34 | 1.22 | 724.08 |
| TDr8902157_81 | 1.12 | 2138.54 | TDr9518988_35 | 1.30 | 759.92 |
| TDr8902157_82 | 1.36 | 660.46 | TDr9518988_36 | 1.40 | 726.20 |
| TDr8902157_84 | 1.21 | 2083.74 | TDr9518988_39 | 1.22 | 1292.16 |
| TDr8902157_87 | 1.21 | 1833.52 | TDr9518988_4 | 1.25 | 597.76 |
| TDr8902157_88 | 1.33 | 680.46 | TDr9518988_40 | 1.37 | 760.64 |
| TDr8902157_89 | 1.16 | 661.25 | TDr9518988_41 | 1.19 | 752.38 |
| TDr8902157_91 | 1.20 | 1273.93 | TDr9518988_42 | 1.39 | 751.91 |
| TDr8902157_94 | 1.14 | 1248.75 | TDr9518988_44 | 1.28 | 1309.94 |
| TDr8902157_96 | 1.26 | 687.45 | TDr9518988_46 | 1.40 | 1264.51 |
| TDr8902475 | 1.31 | 672.94 | TDr9518988_48 | 1.27 | 570.24 |
| TDr8902475_100 | 1.36 | 694.16 | TDr9518988_50 | 1.33 | 1289.17 |
| TDr8902475_102 | 1.32 | 537.80 | TDr9518988_51 | 1.26 | 174.92 |
| TDr8902475_104 | 1.29 | 695.11 | TDr9518988_52 | 1.25 | 1301.12 |
| TDr8902475_106 | 1.24 | 2174.42 | TDr9518988_55 | 1.42 | 1245.63 |
| TDr8902475_107 | 1.25 | 2900.45 | TDr9518988_58 | 1.18 | 763.33 |
| TDr8902475_109 | 1.27 | 399.50 | TDr9518988_59 | 1.30 | 1294.47 |
| TDr8902475_113 | 1.21 | 446.25 | TDr9518988_6 | 1.18 | 783.03 |
| TDr8902475_115 | 1.33 | 1225.90 | TDr9518988_60 | 1.44 | 1263.95 |
| TDr8902475_118 | 1.30 | 1494.57 | TDr9518988_62 | 1.25 | 1283.52 |
| TDr8902475_122 | 1.27 | 2040.10 | TDr9518988_63 | 1.31 | 716.640 |
| TDr8902475_127 | 1.19 | 211.65 | TDr9518988_64 | 1.22 | 1558.40 |
| TDr8902475_128 | 1.25 | 673.55 | TDr9518988_7 | 1.15 | 760.48 |
| TDr8902475_130 | 1.25 | 1406.48 | TDr9518988_70 | 1.17 | 720.73 |
| TDr8902475_54 | 1.27 | 2100.38 | TDr9518988_71 | 1.34 | 1257.14 |
| TDr8902475_63 | 1.28 | 2083.13 | TDr9518988_72 | 1.43 | 1277.44 |
| TDr8902475_94 | 1.14 | 1268.44 | TDr9518988_73 | 1.24 | 568.50 |

Table S2. continued

| Genotype | TYP | YMV | Genotype | TYP | YMV |
| --- | --- | --- | --- | --- | --- |
| TDr9518988_74 | 1.23 | 741.97 | TDr9519158_51 | 1.25 | 559.38 |
| TDr9518988_76 | 1.36 | 1280.53 | TDr9519158_52 | 1.32 | 582.92 |
| TDr9518988_77 | 1.30 | 697.58 | TDr9519158_53 | 1.22 | 755.66 |
| TDr9518988_78 | 1.26 | 1295.17 | TDr9519158_55 | 1.24 | 780.08 |
| TDr9518988_79 | 1.27 | 1248.27 | TDr9519158_56 | 1.12 | 596.47 |
| TDr9518988_8 | 1.35 | 745.94 | TDr9519158_7 | 1.23 | 727.35 |
| TDr9518988_80 | 1.29 | 731.44 | TDr9519177 | 1.14 | 1133.93 |
| TDr9518988_81 | 1.27 | 729.48 | TDr9600629 | 0.95 | 1291.42 |
| TDr9518988_82 | 1.38 | 728.67 | TDr9700205 | 0.97 | 1159.49 |
| TDr9518988_83 | 1.27 | 740.35 | TDr9700205_104 | 1.20 | 677.79 |
| TDr9518988_9 | 1.44 | 710.50 | TDr9700205_106 | 1.26 | 2121.19 |
| TDr9519156 | 1.06 | 1279.66 | TDr9700205_111 | 1.18 | 712.41 |
| TDr9519158 | 1.36 | 885.31 | TDr9700205_112 | 1.10 | 690.47 |
| TDr9519158_11 | 1.27 | 763.33 | TDr9700205_150 | 1.20 | 535.47 |
| TDr9519158_12 | 1.03 | 739.18 | TDr9700205_153 | 1.08 | 701.71 |
| TDr9519158_13 | 1.20 | 738.03 | TDr9700205_158 | 1.14 | 708.51 |
| TDr9519158_14 | 1.32 | 1296.59 | TDr9700205_177 | 1.13 | 1579.13 |
| TDr9519158_16 | 1.19 | 725.54 | TDr9700205_181 | 1.12 | 1559.71 |
| TDr9519158_17 | 1.15 | 1304.54 | TDr9700205_182 | 1.08 | 680.49 |
| TDr9519158_18 | 1.24 | 754.44 | TDr9700205_186 | 1.17 | 707.72 |
| TDr9519158_19 | 1.22 | 748.23 | TDr9700205_196 | 1.18 | 711.35 |
| TDr9519158_2 | 1.23 | 567.20 | TDr9700205_209 | 1.17 | 714.86 |
| TDr9519158_23 | 1.26 | 743.86 | TDr9700205_211 | 1.14 | 693.58 |
| TDr9519158_24 | 1.19 | 748.66 | TDr9700205_216 | 1.06 | 695.82 |
| TDr9519158_27 | 1.22 | 604.89 | TDr9700205_223 | 1.18 | 120.65 |
| TDr9519158_28 | 1.18 | 578.89 | TDr9700205_225 | 1.14 | 719.58 |
| TDr9519158_3 | 1.16 | 768.68 | TDr9700205_227 | 1.19 | 1557.84 |
| TDr9519158_30 | 1.23 | 739.43 | TDr9700205_231 | 1.28 | 695.96 |
| TDr9519158_31 | 1.27 | 762.38 | TDr9700205_234 | 1.19 | 711.26 |
| TDr9519158_33 | 1.12 | 736.95 | TDr9700205_238 | 1.16 | 144.01 |
| TDr9519158_34 | 1.23 | 187.56 | TDr9700205_24 | 1.13 | 707.38 |
| TDr9519158_35 | 1.33 | 787.03 | TDr9700205_247 | 1.15 | 688.42 |
| TDr9519158_36 | 1.24 | 590.08 | TDr9700205_249 | 1.13 | 1284.75 |
| TDr9519158_38 | 1.20 | 743.24 | TDr9700205_253 | 1.09 | 719.26 |
| TDr9519158_39 | 1.24 | 614.92 | TDr9700205_256 | 1.13 | 697.21 |
| TDr9519158_4 | 1.23 | 732.09 | TDr9700205_257 | 1.06 | 2121.51 |
| TDr9519158_40 | 1.23 | 735.65 | TDr9700205_259 | 1.16 | 708.08 |
| TDr9519158_41 | 1.26 | 559.64 | TDr9700205_28 | 1.13 | 429.21 |
| TDr9519158_42 | 1.20 | 754.79 | TDr9700205_30 | 1.04 | 700.92 |
| TDr9519158_43 | 1.18 | 739.83 | TDr9700205_31 | 1.26 | 526.37 |
| TDr9519158_46 | 1.23 | 702.22 | TDr9700205_32 | 1.10 | 1283.85 |
| TDr9519158_48 | 1.23 | 618.18 | TDr9700205_42 | 1.19 | 691.25 |
| TDr9519158_50 | 1.30 | 717.74 | TDr9700632 | 0.98 | 1356.85 |

Table S2. continued

| Genotype | TYP | YMV | Genotype | TYP | YMV |
| --- | --- | --- | --- | --- | --- |
| TDr9700632_11 | 1.15 | 554.14 | TDr9700793_107 | 0.98 | 725.98 |
| TDr9700632_12 | 1.19 | 544.34 | TDr9700793_11 | 1.10 | 721.00 |
| TDr9700632_16 | 1.20 | 536.57 | TDr9700793_123 | 1.16 | 1299.94 |
| TDr9700632_2 | 1.17 | 559.50 | TDr9700793_124 | 1.14 | 165.78 |
| TDr9700632_21 | 1.10 | 714.89 | TDr9700793_125 | 1.14 | 140.2 |
| TDr9700632_23 | 1.09 | 710.02 | TDr9700793_127 | 1.08 | 747.08 |
| TDr9700632_25 | 1.01 | 149.56 | TDr9700793_13 | 1.00 | 1269.61 |
| TDr9700632_29 | 1.14 | 694.99 | TDr9700793_133 | 1.04 | 739.09 |
| TDr9700632_3 | 1.04 | 1561.44 | TDr9700793_136 | 1.15 | 728.59 |
| TDr9700632_30 | 1.22 | 1263.00 | TDr9700793_137 | 1.08 | 746.49 |
| TDr9700632_31 | 1.08 | 732.73 | TDr9700793_138 | 1.16 | 466.91 |
| TDr9700632_33 | 1.12 | 567.21 | TDr9700793_139 | 1.04 | 707.17 |
| TDr9700632_34 | 1.18 | 139.49 | TDr9700793_143 | 0.98 | 708.07 |
| TDr9700632_35 | 1.12 | 163.43 | TDr9700793_145 | 1.01 | 721.86 |
| TDr9700632_36 | 1.23 | 544.49 | TDr9700793_146 | 1.12 | 727.14 |
| TDr9700632_4 | 1.15 | 531.27 | TDr9700793_147 | 1.21 | 605.57 |
| TDr9700632_41 | 1.02 | 122.56 | TDr9700793_148 | 1.00 | 731.34 |
| TDr9700632_42 | 1.16 | 155.53 | TDr9700793_15 | 1.20 | 1539.80 |
| TDr9700632_44 | 1.09 | 1290.40 | TDr9700793_151 | 1.10 | 749.26 |
| TDr9700632_45 | 1.32 | 711.97 | TDr9700793_155 | 1.14 | 2149.43 |
| TDr9700632_48 | 1.12 | 527.40 | TDr9700793_156 | 1.09 | 1287.83 |
| TDr9700632_51 | 1.19 | 530.20 | TDr9700793_159 | 1.17 | 100.56 |
| TDr9700632_53 | 1.14 | 702.85 | TDr9700793_16 | 1.09 | 1575.93 |
| TDr9700632_54 | 1.06 | 1294.01 | TDr9700793_160 | 1.00 | 749.36 |
| TDr9700632_58 | 1.12 | 558.51 | TDr9700793_163 | 1.10 | 708.04 |
| TDr9700632_59 | 1.15 | 730.84 | TDr9700793_18 | 1.11 | 795.45 |
| TDr9700632_6 | 1.05 | 2144.75 | TDr9700793_20 | 1.20 | 745.56 |
| TDr9700632_60 | 1.20 | 557.50 | TDr9700793_21 | 1.04 | 720.45 |
| TDr9700632_63 | 1.17 | 580.08 | TDr9700793_22 | 0.97 | 2156.84 |
| TDr9700632_67 | 1.16 | 693.05 | TDr9700793_23 | 0.98 | 876.82 |
| TDr9700632_69 | 1.25 | 709.63 | TDr9700793_24 | 0.98 | 2021.38 |
| TDr9700632_70 | 1.06 | 718.89 | TDr9700793_25 | 1.10 | 1569.21 |
| TDr9700632_72 | 1.20 | 526.09 | TDr9700793_26 | 1.09 | 1305.61 |
| TDr9700632_77 | 1.17 | 557.52 | TDr9700793_29 | 1.18 | 1590.84 |
| TDr9700632_8 | 1.12 | 150.71 | TDr9700793_33 | 1.17 | 1595.10 |
| TDr9700632_83 | 1.08 | 1302.58 | TDr9700793_35 | 0.96 | 1008.59 |
| TDr9700632_84 | 1.15 | 726.12 | TDr9700793_36 | 1.12 | 778.52 |
| TDr9700632_86 | 1.21 | 542.21 | TDr9700793_37 | 1.15 | 1269.92 |
| TDr9700632_87 | 1.16 | 544.07 | TDr9700793_38 | 0.97 | 755.97 |
| TDr9700793_1 | 1.12 | 1572.24 | TDr9700793_5 | 1.11 | 2390.16 |
| TDr9700793_101 | 1.02 | 749.18 | TDr9700793_51 | 0.99 | 734.40 |
| TDr9700793_102 | 1.00 | 739.39 | TDr9700793_71 | 1.10 | 721.28 |
| TDr9700793_106 | 0.97 | 102.50 | TDr9700793_8 | 1.03 | 707.96 |

Table S2. continued

| Genotype | TYP | YMV | Genotypes | TYP | YMV |
| --- | --- | --- | --- | --- | --- |
| TDr9700917 | 1.22 | 1091.45 | TDrOjuiyawo_171 | 1.27 | 133.67 |
| TDr9902562 | 1.29 | 911.38 | TDrOjuiyawo_173 | 1.30 | 677.14 |
| TDr9902626 | 1.14 | 1335.81 | TDrOjuiyawo_176 | 1.32 | 640.13 |
| TDr9902789 | 1.15 | 1376.09 | TDrOjuiyawo_177 | 1.29 | 648.50 |
| TDrAgbanwobe | 0.93 | 1371.87 | TDrOjuiyawo_185 | 1.37 | 653.27 |
| TDrAlumaco | 1.05 | 925.74 | TDrOjuiyawo_19 | 1.16 | 2075.47 |
| TDrDanacha | 1.07 | 781.15 | TDrOjuiyawo_33 | 1.24 | 699.18 |
| TDrEhobia | 1.09 | 1089.51 | TDrOjuiyawo_35 | 1.21 | 759.38 |
| TDrFakesta | 1.33 | 931.19 | TDrOjuiyawo_39 | 1.35 | 675.66 |
| TDrLeusi | 1.33 | 828.20 | TDrOjuiyawo_52 | 1.36 | 703.61 |
| TDrMeccakusa | 1.34 | 717.39 | TDrOjuiyawo_53 | 1.11 | 2114.82 |
| TDrNndu | 1.47 | 739.47 | TDrOjuiyawo_54 | 1.47 | 1228.08 |
| TDrOjuiyawo | 1.20 | 1037.61 | TDrOjuiyawo_56 | 1.15 | 720.02 |
| TDrOjuiyawo_108 | 1.38 | 1517.26 | TDrOjuiyawo_60 | 1.24 | 2105.97 |
| TDrOjuiyawo_113 | 1.14 | 692.37 | TDrOjuiyawo_61 | 1.29 | 656.39 |
| TDrOjuiyawo_116 | 1.41 | 676.52 | TDrOjuiyawo_64 | 1.21 | 1237.40 |
| TDrOjuiyawo_129 | 1.30 | 1815.75 | TDrOjuiyawo_67 | 1.18 | 2678.80 |
| TDrOjuiyawo_132 | 1.23 | 1528.80 | TDrOjuiyawo_71 | 1.18 | 736.66 |
| TDrOjuiyawo_143 | 1.32 | 677.92 | TDrOjuiyawo_75 | 1.28 | 543.88 |
| TDrOjuiyawo_145 | 1.21 | 668.53 | TDrOjuiyawo_81 | 1.28 | 1254.21 |
| TDrOjuiyawo_146 | 1.12 | 2086.20 | TDrOjuiyawo_82 | 1.11 | 2092.48 |
| TDrOjuiyawo_150 | 1.34 | 2077.87 | TDrOjuiyawo_85 | 1.28 | 529.45 |
| TDrOjuiyawo_155 | 1.14 | 2105.70 | TDrOjuiyawo_88 | 1.39 | 686.98 |
| TDrOjuiyawo_157 | 1.30 | 684.20 | TDrOjuiyawo_89 | 1.37 | 698.16 |
| TDrOjuiyawo_159 | 1.25 | 1251.20 | TDrOjuiyawo_91 | 1.35 | 702.67 |
| TDrOjuiyawo_160 | 1.25 | 1519.50 | TDrOjuiyawo_96 | 1.16 | 718.42 |
| TDrOjuiyawo_162 | 1.36 | 689.65 | TDrOjuiyawo_97 | 1.22 | 2126.68 |
| TDrOjuiyawo_163 | 1.19 | 1240.99 | TDrPampers | 1.36 | 798.81 |
| TDrOjuiyawo_167 | 1.28 | 670.80 | TDrPouna | 1.34 | 1041.11 |
| TDrOjuiyawo_169 | 1.18 | 1812.18 | TDrUfenyi | 1.07 | 1285.55 |
| TDrOjuiyawo_17 | 1.20 | 698.26 | TDrYangbedu | 0.97 | 951.16 |

Table S3. Cluster membership of 406 genotypes of white yam based phylogeny clustering

| **Genotypes** | **Cluster** | **Genotypes** | **Cluster** | **Genotypes** | **Cluster** |
| --- | --- | --- | --- | --- | --- |
| TDr8902475 | 1 | TDrOjuiyawo_160 | 1 | TDr1100734 | 1 |
| TDr9518988_44 | 1 | TDr1603_5 | 1 | TDr1000605 | 1 |
| TDr9518988_62 | 1 | TDr9519158_56 | 1 | TDr9518988_34 | 2 |
| TDr8902475_99 | 1 | TDrYangbedu | 1 | TDr9518988_59 | 2 |
| TDr8902475_113 | 1 | TDrOjuiyawo_53 | 1 | TDr8902475_54 | 2 |
| TDr8902475_94 | 1 | TDrOjuiyawo_75 | 1 | TDr8902475_106 | 2 |
| TDr9518988_58 | 1 | TDrOjuiyawo_96 | 1 | TDr8902475_122 | 2 |
| TDr9518988 | 1 | TDr1606_6 | 1 | TDr9518988_14 | 2 |
| TDr9902789 | 1 | TDrOjuiyawo_54 | 1 | TDr9518988_35 | 2 |
| TDr9700205 | 1 | TDrOjuiyawo_81 | 1 | TDr9518988_46 | 2 |
| TDr8902157_4 | 1 | TDrOjuiyawo_97 | 1 | TDr9518988_60 | 2 |
| TDr8902157_49 | 1 | TDrOjuiyawo_146 | 1 | TDr8902475_98 | 2 |
| TDr8902157_67 | 1 | TDr1602_4 | 1 | TDr8902475_107 | 2 |
| TDr8902157_78 | 1 | TDr9902626 | 1 | TDr9518988_17 | 2 |
| TDr8902157 | 1 | TDrOjuiyawo_82 | 1 | TDr9518988_36 | 2 |
| TDr8902157_8 | 1 | TDrOjuiyawo_108 | 1 | TDr9518988_48 | 2 |
| TDr8902157_57 | 1 | TDr1602_5 | 1 | TDr8902475_109 | 2 |
| TDr8902157_69 | 1 | TDr1606_16 | 1 | TDr8902475_127 | 2 |
| TDr8902157_79 | 1 | TDrUfenyi | 1 | TDr9518988_4 | 2 |
| TDr8902157_91 | 1 | TDrOjuiyawo_60 | 1 | TDr9518988_50 | 2 |
| TDr8902157_55 | 1 | TDrOjuiyawo_85 | 1 | TDr9518988_63 | 2 |
| TDr8902157_16 | 1 | TDrOjuiyawo_113 | 1 | TDr8902475_100 | 2 |
| TDr8902157_37 | 1 | TDrOjuiyawo_169 | 1 | TDr8902475_128 | 2 |
| TDr8902157_58 | 1 | TDrOjuiyawo_61 | 1 | TDr9518988_6 | 2 |
| TDr8902157_72 | 1 | TDrOjuiyawo_88 | 1 | TDr9518988_20 | 2 |
| TDr8902157_81 | 1 | TDrOjuiyawo_155 | 1 | TDr9518988_39 | 2 |
| TDr8902157_94 | 1 | TDrOjuiyawo_171 | 1 | TDr9518988_51 | 2 |
| TDr8902157_73 | 1 | TDrOjuiyawo_35 | 1 | TDr8902475_63 | 2 |
| TDr8902157_1 | 1 | TDrOjuiyawo_89 | 1 | TDr8902475_102 | 2 |
| TDr8902157_64 | 1 | TDrOjuiyawo_173 | 1 | TDr8902475_115 | 2 |
| TDr8902157_103 | 1 | TDr1603_2 | 1 | TDr8902475_130 | 2 |
| TDr8902157_24 | 1 | TDr9600629 | 1 | TDr9518988_7 | 2 |
| TDr8902157_76 | 1 | TDr1000245 | 1 | TDr9518988_23 | 2 |
| TDr8902157_87 | 1 | TDrPouna | 1 | TDr9518988_40 | 2 |
| TDr8902157_3 | 1 | TDrFakesta | 1 | TDr9518988_52 | 2 |
| TDr8902157_48 | 1 | TDr0900013 | 1 | TDr9518988_27 | 2 |
| TDr8902157_66 | 1 | TDr8902677 | 1 | TDr9518988_8 | 2 |
| TDr9519158_12 | 1 | TDr04_219 | 1 | TDr9518988_24 | 2 |
| TDrOjuiyawo_39 | 1 | TDrMeccakusa | 1 | TDr9518988_41 | 2 |
| TDrOjuiyawo_67 | 1 | TDr06_15 | 1 | TDr9518988_55 | 2 |
| TDrOjuiyawo_159 | 1 | TDrPampers | 1 | TDr8902475_104 | 2 |
| TDr1603_3 | 1 | TDr1100180 | 1 | TDr8902475_118 | 2 |
| TDrOjuiyawo_71 | 1 | TDrLeusi | 1 | TDr9518988_9 | 2 |

Table S3. Continued

| **Genotypes** | **Cluster** | **Genotypes** | **Cluster** | **Genotypes** | **Cluster** |
| --- | --- | --- | --- | --- | --- |
| TDr9518988_28 | 2 | TDrOjuiyawo_116 | 2 | TDr1100015 | 3 |
| TDr9518988_42 | 2 | TDr1609_14 | 2 | TDr1101408 | 3 |
| TDr9518988_12 | 2 | TDrOjuiyawo_64 | 2 | TDr1000144 | 3 |
| TDr9518988_30 | 2 | TDrOjuiyawo_129 | 2 | TDr1100629 | 3 |
| TDr9518988_64 | 2 | TDrOjuiyawo_157 | 2 | TDrAgbanwobe | 3 |
| TDr9518988_74 | 2 | TDr8902665 | 3 | TDr0900152 | 3 |
| TDr9518988_83 | 2 | TDr9700205_32 | 3 | TDrOjuiyawo | 4 |
| TDr8902157_30 | 2 | TDr9700205_28 | 3 | TDr8902157_89 | 4 |
| TDr9518988_76 | 2 | TDr8902157_74 | 3 | TDr9700793_160 | 4 |
| TDr9518988_77 | 2 | TDr9700205_30 | 3 | TDr9700793_102 | 4 |
| TDr9518988_78 | 2 | TDr9700205_227 | 3 | TDr9700793_136 | 4 |
| TDr9518988_70 | 2 | TDr9700205_153 | 3 | TDr9700793_148 | 4 |
| TDr9518988_79 | 2 | TDr9700205_231 | 3 | TDr9700793_163 | 4 |
| TDr8902157_62 | 2 | TDr9700205_257 | 3 | TDr9700793_21 | 4 |
| TDr9518988_71 | 2 | TDr9700205_216 | 3 | TDr9700793_35 | 4 |
| TDr9518988_80 | 2 | TDr9700205_111 | 3 | TDr9700793_106 | 4 |
| TDr8902157_84 | 2 | TDr9700205_223 | 3 | TDr9700793_8 | 4 |
| TDr9518988_72 | 2 | TDr9700632 | 3 | TDr9700793_22 | 4 |
| TDr9518988_81 | 2 | TDr9700632_30 | 3 | TDr9700793_107 | 4 |
| TDr8902157_2 | 2 | TDr9700632_53 | 3 | TDr9700793_11 | 4 |
| TDr8902157_65 | 2 | TDr9700632_69 | 3 | TDr9700793_23 | 4 |
| TDr9518988_73 | 2 | TDr9700632_2 | 3 | TDr9700793_139 | 4 |
| TDr9518988_82 | 2 | TDr9700632_44 | 3 | TDr9700793_13 | 4 |
| TDr9519158_35 | 2 | TDr9700632_3 | 3 | TDr9700793_24 | 4 |
| TDr1609_29 | 2 | TDr9700632_72 | 3 | TDr9700793_38 | 4 |
| TDrOjuiyawo_91 | 2 | TDr9700632_48 | 3 | TDr9700793_143 | 4 |
| TDrOjuiyawo_132 | 2 | TDr9700632_4 | 3 | TDr9700793_51 | 4 |
| TDrOjuiyawo_176 | 2 | TDr9700632_21 | 3 | TDr9700793_145 | 4 |
| TDr1609_30 | 2 | TDr9700632_58 | 3 | TDr9700793_26 | 4 |
| TDrOjuiyawo_52 | 2 | TDr9700632_77 | 3 | TDr9700793_125 | 4 |
| TDrOjuiyawo_143 | 2 | TDr9700632_59 | 3 | TDr9700793_146 | 4 |
| TDrOjuiyawo_177 | 2 | TDr9700632_83 | 3 | TDr1601_11 | 4 |
| TDrOjuiyawo_145 | 2 | TDr9700632_60 | 3 | TDr1601_12 | 4 |
| TDrOjuiyawo_167 | 2 | TDr9700632_84 | 3 | TDrOjuiyawo_163 | 4 |
| TDrOjuiyawo_17 | 2 | TDr9700632_25 | 3 | TDr1601_9 | 4 |
| TDr1608_24 | 2 | TDr9700632_51 | 3 | TDr9518544 | 4 |
| TDrOjuiyawo_56 | 2 | TDr9700632_63 | 3 | TDr1101272 | 4 |
| TDrOjuiyawo_150 | 2 | TDr9700632_86 | 3 | TDr0000362 | 4 |
| TDrOjuiyawo_185 | 2 | TDr9700632_41 | 3 | TDr9700917 | 4 |
| TDrOjuiyawo_19 | 2 | TDr9700632_67 | 3 | TDr9519156 | 4 |
| TDr1608_36 | 2 | TDr1604_56 | 3 | TDrNndu | 4 |
| TDrOjuiyawo_162 | 2 | TDr1604_58 | 3 | TDrDanacha | 4 |
| TDrOjuiyawo_33 | 2 | TDr1604_60 | 3 | TDr1101142 | 4 |

Table S3. Continued

| **Genotypes** | **Cluster** | **Genotypes** | **Cluster** | **Genotypes** | **Cluster** |
| --- | --- | --- | --- | --- | --- |
| TDrAlumaco | 4 | TDr9519158_42 | 5 | TDr9700205_247 | 6 |
| TDr1100291 | 4 | TDr9519158_3 | 5 | TDr9700205_106 | 6 |
| TDr9519177 | 4 | TDr9519158_24 | 5 | TDr9700205_249 | 6 |
| TDrEhobia | 4 | TDr9519158_43 | 5 | TDr9700205_181 | 6 |
| TDr1000600 | 4 | TDr9519158_4 | 5 | TDr9700632_16 | 6 |
| TDr9519158 | 5 | TDr9519158_13 | 5 | TDr9700632_42 | 6 |
| TDr9501932 | 5 | TDr9519158_55 | 5 | TDr9700632_31 | 6 |
| TDr8902157_32 | 5 | TDr1607_10 | 5 | TDr9700632_54 | 6 |
| TDr8902157_71 | 5 | TDr1000282 | 5 | TDr9700632_70 | 6 |
| TDr8902157_17 | 5 | TDr1100128 | 5 | TDr9700632_33 | 6 |
| TDr8902157_82 | 5 | TDr1100228 | 5 | TDr9700632_45 | 6 |
| TDr8902157_96 | 5 | TDr0900263 | 5 | TDr9700632_34 | 6 |
| TDr8902157_43 | 5 | TDr1101701 | 5 | TDr9700632_6 | 6 |
| TDr8902157_45 | 5 | TDr1000563 | 5 | TDr9700632_23 | 6 |
| TDr8902157_29 | 5 | TDr1000021 | 5 | TDr9700632_35 | 6 |
| TDr8902157_77 | 5 | TDr1000248 | 5 | TDr9700632_8 | 6 |
| TDr8902157_88 | 5 | TDr0900135 | 5 | TDr9700632_36 | 6 |
| TDr9519158_27 | 5 | TDr9902562 | 5 | TDr9700632_11 | 6 |
| TDr9519158_36 | 5 | TDr1100008 | 5 | TDr9700632_12 | 6 |
| TDr9519158_46 | 5 | TDr1000149 | 5 | TDr9700632_29 | 6 |
| TDr9519158_14 | 5 | TDr1000052 | 5 | TDr9700632_87 | 6 |
| TDr9519158_28 | 5 | TDr1000060 | 5 | TDr9700793_133 | 7 |
| TDr9519158_48 | 5 | TDr1000310 | 5 | TDr9700793_147 | 7 |
| TDr9519158_7 | 5 | TDr0902079 | 5 | TDr9700793_1 | 7 |
| TDr9519158_16 | 5 | TDr9700205_42 | 6 | TDr9700793_20 | 7 |
| TDr9519158_30 | 5 | TDr9700205_24 | 6 | TDr9700793_33 | 7 |
| TDr9519158_38 | 5 | TDr9700205_31 | 6 | TDr9700793_5 | 7 |
| TDr9519158_50 | 5 | TDr9700205_112 | 6 | TDr9700793_137 | 7 |
| TDr9519158_17 | 5 | TDr9700205_182 | 6 | TDr9700793_151 | 7 |
| TDr9519158_31 | 5 | TDr9700205_225 | 6 | TDr9700793_36 | 7 |
| TDr9519158_39 | 5 | TDr9700205_253 | 6 | TDr9700793_138 | 7 |
| TDr9519158_51 | 5 | TDr9700205_150 | 6 | TDr9700793_37 | 7 |
| TDr9519158_18 | 5 | TDr9700205_186 | 6 | TDr9700793_155 | 7 |
| TDr9519158_40 | 5 | TDr9700205_256 | 6 | TDr9700793_123 | 7 |
| TDr9519158_52 | 5 | TDr9700205_196 | 6 | TDr9700793_156 | 7 |
| TDr9519158_19 | 5 | TDr9700205_158 | 6 | TDr9700793_15 | 7 |
| TDr9519158_33 | 5 | TDr9700205_209 | 6 | TDr9700793_25 | 7 |
| TDr9519158_41 | 5 | TDr9700205_234 | 6 | TDr9700793_124 | 7 |
| TDr9519158_53 | 5 | TDr9700205_259 | 6 | TDr9700793_16 | 7 |
| TDr9519158_2 | 5 | TDr9700205_211 | 6 | TDr9700793_71 | 7 |
| TDr9519158_11 | 5 | TDr9700205_238 | 6 | TDr9700793_159 | 7 |
| TDr9519158_23 | 5 | TDr9700205_104 | 6 | TDr9700793_18 | 7 |
| TDr9519158_34 | 5 | TDr9700205_177 | 6 | TDr9700793_29 | 7 |

Table S3. continued

| **Genotypes** | **Cluster** | **Genotypes** | **Cluster** | **Genotypes** | **Cluster** |
| --- | --- | --- | --- | --- | --- |
| TDr9700793_101 | 7 | TDr0900220 | 7 | TDr0900121 | 7 |
| TDr9700793_127 | 7 | TDr1000412 | 7 | TDr1000228 | 7 |
| TDr0900341 | 7 | TDr0900122 | 7 | TDr0900295 | 7 |
| TDr1001012 | 7 | TDr8900983 | 7 | TDr1100055 | 7 |
| TDr0900267 | 7 | TDr0900408 | 7 | TDr0900134 | 7 |
| TDr0000001 | 7 | TDr0900404 | 7 | TDr0900052 | 7 |

Table S4. Single nucleotide polymorphism (SNP) markers associated with the yield per plant (TYP) and yam mosaic virus (YMV) and putative genes identified in chromosomes of 406 clones of white yam

| Trait | SNP | Chr. | Position (bp) | Start | End | Putative genes identified |
| --- | --- | --- | --- | --- | --- | --- |
| TYP | chr04_6236404 | 4 | 23401186 | IPR001128 | IPR001245 | Gibberellin regulated protein (IPR022692); AP2/ERF domain (IPR001471), NB-ARC (IPR002182); Dirigent protein (IPR004265); Membrane transport protein (IPR004776), Importin subunit beta-1, plants (IPRO27140) |
|  | chr04_8196378 | 4 | 8196378 | IPR001128 | IPR001245 | Gibberellin regulated protein (IPR022692); AP2/ERF domain (IPR001471), NB-ARC (IPR002182); Dirigent protein (IPR004265); Membrane transport protein (IPR004776), Importin subunit beta-1, plants (IPRO27140) |
|  | chr04_18269860 | 4 | 18269860 | IPR001128 | IPR001245 | Gibberellin regulated protein (IPR022692); AP2/ERF domain (IPR001471), NB-ARC (IPR002182); Dirigent protein (IPR004265); Membrane transport protein (IPR004776), Importin subunit beta-1, plants (IPRO27140) |
|  | chr04_23401186 | 4 | 6236404 | IPR001128 | IPR001245 | Gibberellin regulated protein (IPR022692); AP2/ERF domain (IPR001471), NB-ARC (IPR002182); Dirigent protein (IPR004265); Membrane transport protein (IPR004776), Importin subunit beta-1, plants (IPRO27140) |
|  | chr05_24237388 | 5 | 24237388 | IPR003441 | IPR022742 | Expansin (IPR002963); AUX/IAA protein (IPR003311); AP2/ERF domain (IPR001471) |
|  | chr05_24682916 | 5 | 24682916 | IPR003441 | IPR022742 | Expansin (IPR002963); AUX/IAA protein (IPR003311); AP2/ERF domain (IPR001471) |
|  | chr08_7046574 | 8 | 7046574 | IPR000916 | IPR010678 | AUX/IAA protein (IPR003311); Glycine rich protein (IPR010800); Protein ENHANCED DISEASE RESISTANCE 2, C-terminal (IPR009769) |
|  | chr08_10135940 | 8 | 10135940 | IPR000916 | IPR010678 | AUX/IAA protein (IPR003311); Glycine rich protein (IPR010800); Protein ENHANCED DISEASE RESISTANCE 2, C-terminal (IPR009769) |
|  | chr10_1317508 | 10 | 1571815 | IPR011333 | IPR002836 | AP2/ERF domain (IPR001471) |
|  | chr10_1571815 | 10 | 1317508 | IPR011333 | IPR002836 | AP2/ERF domain (IPR001471) |
|  | chr13_13467988 | 13 | 13467988 | IPR001085 | IPR023210 | AP2/ERF domain (IPR001471); NB-ARC (IPR002182); AUX/IAA protein (IPR003311); Mo25-like (IPR013878); Major facilitator, sugar transporter-like (IPR005828) |
|  | chr14_11301309 | 14 | 11301309 | IPR004158 | IPR003676 | Expansin, cellulose-binding-like domain (IPR007117); Mitochondrial substrate/solute carrier (IPR018108); Expansin (IPR002963); Root cap (IPR009646); Dirigent protein (IPR004265); Small auxin-up RNA (IPR003676); Major facilitator, sugar transporter-like (IPR005828) |

TYP: tuber yield (kg plant^-1^), YMV: Yam mosaic virus severity score (AUDPC value); Chr.: Chromosome; bp: base pair

Table S4. Continued

| Trait | SNP | Chr. | Position (bp) | Start | End | Putative genes identified |
| --- | --- | --- | --- | --- | --- | --- |
| TYP | chr14_11128124 | 14 | 11128124 | IPR004158 | IPR003676 | Expansin, cellulose-binding-like domain (IPR007117); Mitochondrial substrate/solute carrier (IPR018108); Expansin (IPR002963); Root cap (IPR009646); Dirigent protein (IPR004265); Small auxin-up RNA (IPR003676); Major facilitator, sugar transporter-like (IPR005828) |
|  | chr15_5858214 | 15 | 5858214 | IPR011545 | IPR003439 | Gibberellin regulated protein (IPR003854); Major facilitator, sugar transporter-like (IPR005828); Senescence regulator S40 (IPR007608); ABC transporter-like (IPR003439) |
|  | chr17_15363223 | 17 | 15363223 | IPR001878 | IPR000608 | AUX/IAA protein (IPR003311); ABC transporter-like (IPR003439); Mitochondrial substrate/solute carrier (IPR018108); AP2/ERF domain (IPR001471) |
|  | chr17_19041958 | 17 | 19041958 | IPR001878 | IPR000608 | AUX/IAA protein (IPR003311); ABC transporter-like (IPR003439); Mitochondrial substrate/solute carrier (IPR018108); AP2/ERF domain (IPR001471) |
|  | chr19_9446619 | 19 | 9446619 | IPR013650 | IPR007592 | Exportin-1/Importin-beta-like (IPR013598); Expansin (IPR002963); Sodium/calcium exchanger membrane region (IPR004837); Major facilitator, sugar transporter-like (IPR005828); AUX/IAA protein (IPR003311) |
| YMV | chr03_6338751 | 3 | 6338751 | IPR013128 | IPR009769 | AP2/ERF domain (IPR001471); AUX/IAA protein (IPR003311); Major facilitator, sugar transporter-like (IPR005828) |
|  | chr05_30671001 | 5 | 30671001 | IPR003441 | IPR022742 | Expansin (IPR002963); AUX/IAA protein (IPR003311); AP2/ERF domain (IPR001471) |
|  | chr10_1116193 | 10 | 1116193 | IPR011333 | IPR002836 | [Geminivirus AL1 replication-associated protein, catalytic domain (IPR022690); Geminivirus Rep catalytic domain (PF00799); Geminivirus AL3 coat protein (IPR000657), AP2/ERF domain (IPR001471), NB-ARC (IPR002182); Chlorophyll A-B binding protein, plant and chromista](https://www.ebi.ac.uk/interpro/entry/pfam/PF00799/) (IPR001344) |
|  | chr15_3906069 | 15 | 3906069 | IPR011545 | IPR003439 | AP2/ERF domain (IPR001471); NB-ARC (IPR002182); Gibberellin regulated protein (IPR003854); Major facilitator, sugar transporter-like (IPR005828); ABC transporter-like (IPR003439) |
|  | chr16_1482029 | 16 | 1482029 | IPR015661 | IPR016135 | Geminivirus AR1/BR1 coat protein (IPR000263); AP2/ERF domain (IPR001471); Geminivirus AL1 replication-associated protein, catalytic domain (IPR022690); Geminivirus AL1 replication-associated protein, central domain (IPR022692); NB-ARC (IPR002182) |

TYP: tuber yield (kg plant^-1^), YMV: Yam mosaic virus severity score (AUDPC value); Chr.: Chromosome; bp: base pair
